# Lost bioscapes: Floristic and arthropod diversity coincident with 12^th^ century Polynesian settlement, Nuku Hiva, Marquesas Islands

**DOI:** 10.1371/journal.pone.0265224

**Published:** 2022-03-30

**Authors:** Melinda S. Allen, Tara Lewis, Nick Porch

**Affiliations:** 1 Anthropology, School of Social Sciences, The University of Auckland, Auckland, New Zealand; 2 Te Pūnaha Matatini, Centre of Research Excellence for Complex Systems, The University of Auckland (Host), Auckland, New Zealand; 3 School of Life and Environmental Sciences, Deakin University, Geelong, Australia; New York State Museum, UNITED STATES

## Abstract

Knowledge of biodiversity in the past, and the timing, nature, and drivers of human-induced ecological change, is important for gaining deep time perspectives and for modern conservation efforts. The Marquesas Islands (Polynesia) are one of the world’s most remote archipelagos and illustrate the vulnerability of indigenous bioscapes to anthropogenic activities. Characterised by high levels of endemism across many biotic groups, the full spectrum of the group’s flora and fauna is nonetheless incompletely known. Several centuries of Polynesian settlement reshaped biotic communities in ways that are not yet fully understood, and historically-introduced mammalian herbivores have devastated the indigenous lowland flora. We report here on archaeological recovery of a diverse assemblage of plant and arthropod subfossils from a waterlogged deposit on the largest Marquesan island: Nuku Hiva. These materials offer new perspectives on the composition of lowland plant and arthropod communities pene-contemporaneous with human arrival. Bayesian analysis of multiple ^14^C results from short-lived materials date the assemblages to the mid-12^th^ century AD (*1129–1212 cal*. *AD*, 95.4% HPD). Evidence for human activities in the catchment coincident with deposit formation includes Polynesian associated arthropods, microcharcoal, and an adzed timber. Plant macrofossils (seeds, fruits, vegetative structures) and microfossils (pollen, phytoliths) reveal coastal and lowland wet-moist forest communities unlike those observed today. Several apparently extinct taxa are identified, along with extant taxa currently constrained to high altitude and/or interior areas. A diverse inventory of subfossil arthropods—the first pre-18^th^ century records for the islands—includes more than 100 distinct taxa, with several new archipelago records and one previously unreported for eastern Polynesia. The assemblages provide new insights into lowland Marquesan forest communities coincident with human arrival, and portend the considerable anthropogenic transformations that followed. These records also have implications for human colonisation of the Marquesas Islands and East Polynesia at large.

## Introduction

Human colonists spreading into and across Oceania, transformed island landscapes in the course of creating productive, sustainable socio-natural ecosystems. However, as native vegetation was cleared to make way for gardens and settlements, indigenous/native species were unintentionally extirpated, ecological relations disrupted, and biotic communities reshaped. Knowledge of biodiversity in the past, and understanding the timing, nature, and drivers of human-induced ecological change, is important both for gaining deep time perspectives and for modern conservation and biotic recovery efforts [1–8].

In the Pacific Islands, faunal losses are often attributed to human hunting, especially the extirpation and extinction of flightless birds and large terrestrial reptiles [9–14]. But in many cases significant consequences arguably arose from anthropogenic habitat alteration and translocated predators and competitors [15–20]. In the case of Polynesian colonists, indigenous forest was cleared for cultivation of plant domesticates, often aided by fire, and some indigenous species encouraged at the expense of others [21–26]. These anthropogenic processes fundamentally altered island ecosystems and ecological relationships—on occasion resulting in the complete or near-complete loss of distinctive biotic communities [27–30], with consequential effects for biodiversity [19, 31, 32]. However, the full array of species that accompanied human arrival, the nature of community restructuring that followed, and the long-term consequences for island ecosystems and indigenous taxa are incompletely understood. Transdisciplinary paleoecological partnerships are critical to developing more refined understandings of the ways that human activities have altered biotically isolated and often fragile insular environments.

**Fig 1:**
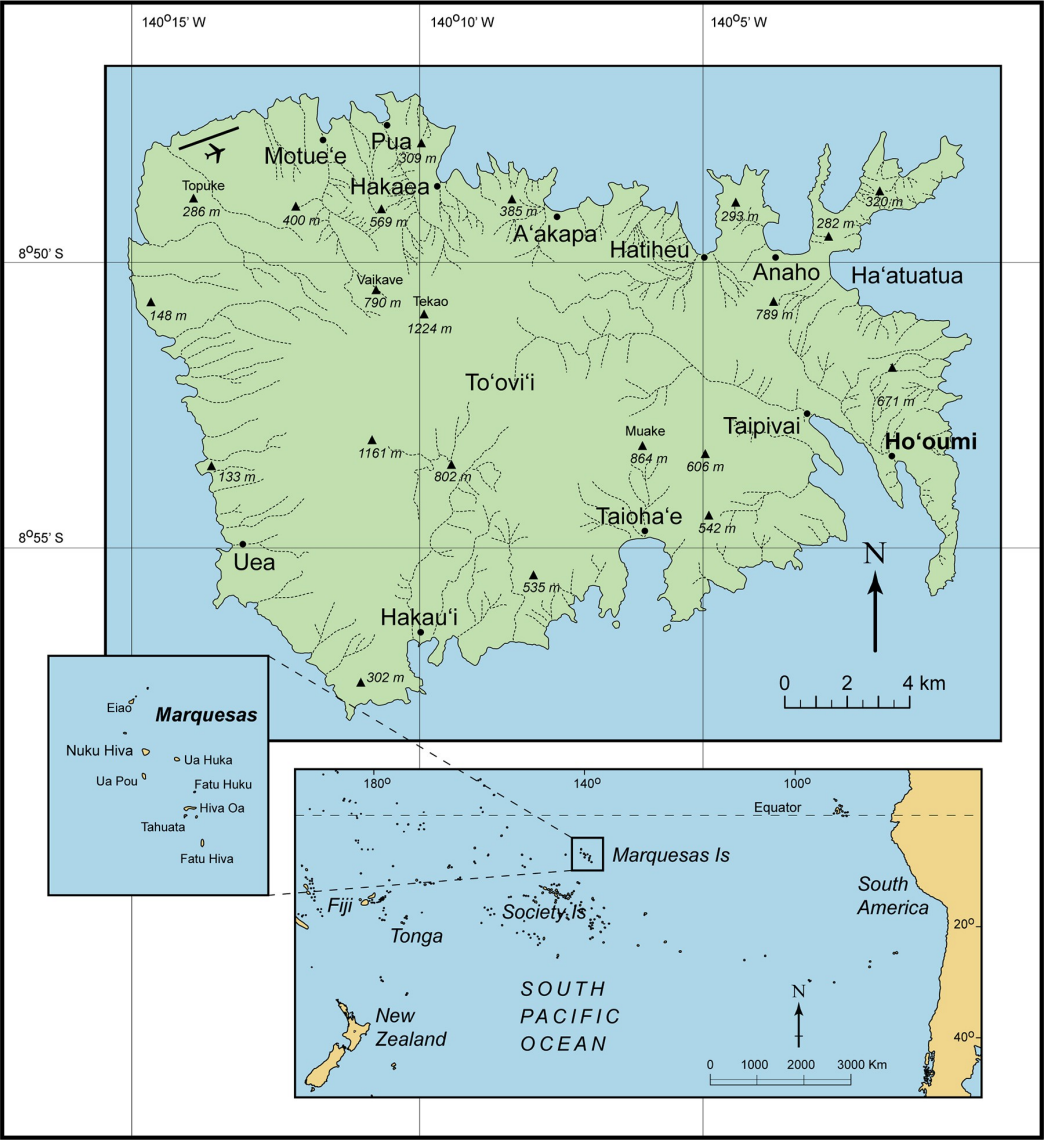
The island of Nuku Hiva, Marquesas Islands, East Polynesia. Ho‘oumi Valley is located on the southeastern coast, adjacent to the large valley of Taipivai.

**Fig 2:**
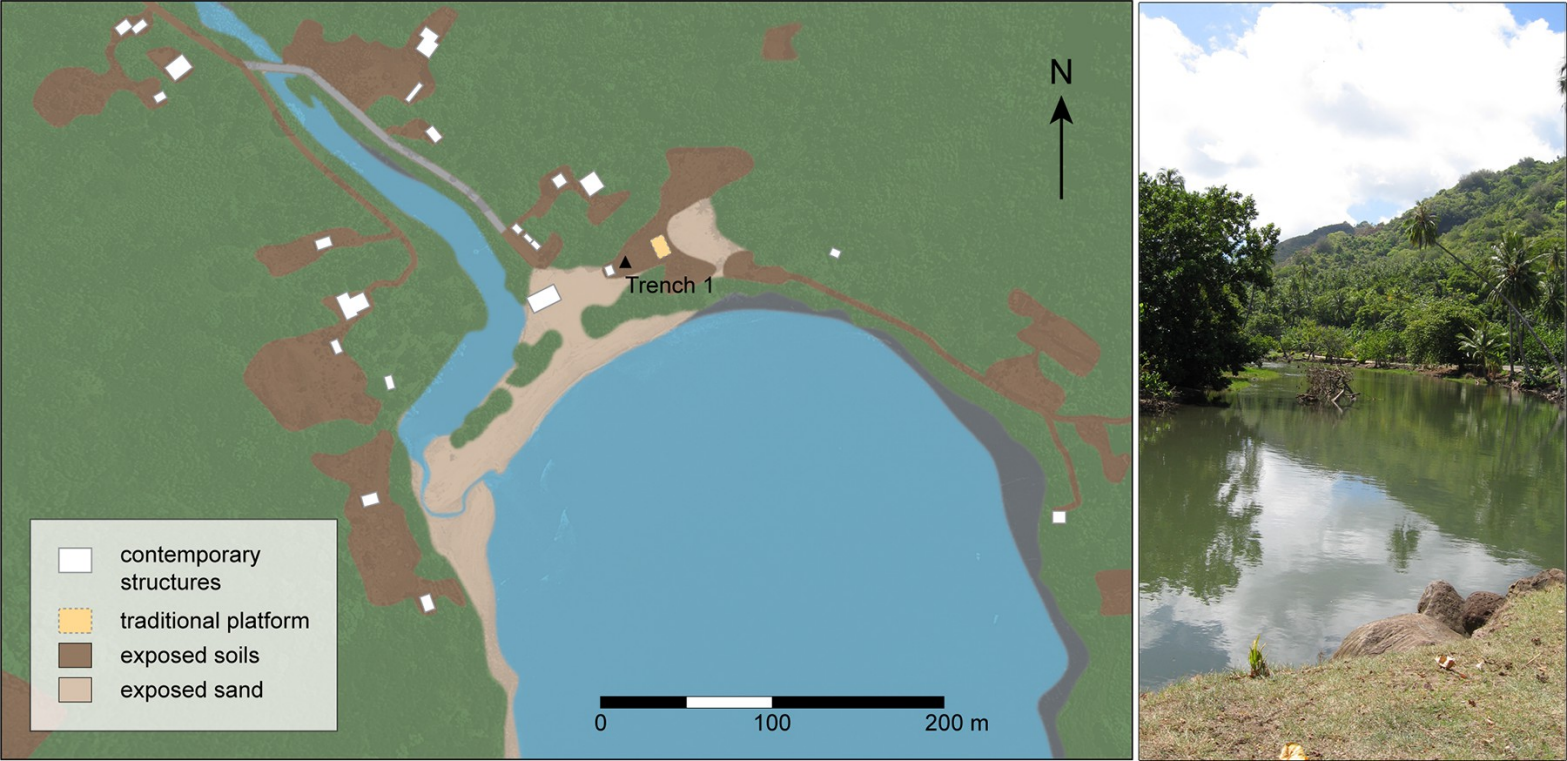
Map of Ho‘oumi Beach showing the location of Trench 1 (left) and an view of the river, looking upstream (right). (Photo by M.S. Allen).

The Marquesas Islands (Fig 1) are a particularly interesting locality in which to examine these processes. Their relative geographic isolation has resulted in a high degree of floral and faunal endemism and many native taxa are now threatened. As with human settlement elsewhere in East Polynesia, colonists arriving in the late Holocene introduced numerous non-native species, including not only domesticated mammals (pig and dog) and the Polynesian chicken, but also the Polynesian Rat (*Rattus exulans*) [20, 33], a commensal lizard (*Lipinia noctua*) [34], and a multitude of useful plants [35–37]. Although early Polynesian arthropod introductions have been identified elsewhere [17, 19], suitable Marquesan deposits have been lacking. Here we report on exceptionally well-preserved plant and arthropod assemblages recovered from Nuku Hiva Island (Fig 2) during the course of archaeological field studies. The biotic remains are mainly comprised of indigenous taxa, but several demonstrable Polynesian introductions are present as well. The findings offer a window on lowland Marquesan bioscapes at or soon after Polynesian arrival, and insights into the earliest human “fingerprints” that preceded the more consequential “footprints” of full-fledged settlement.

## Background

### Marquesan environment and contemporary biota

The Marquesas Archipelago consists of eight main islands located in the central eastern Pacific between 7° and 10° latitude south and approximately 1400 km northeast of the Society Islands. At 340 km^2^, Nuku Hiva (ca. 8°53′S, 140°08′W) is the second largest landmass in the central cultural-historical region of East Polynesia. The island’s topography is rugged, with extensive coastal cliffs, deeply incised valleys, and mountainous ridges rising to 1224 m. The Nuku Hiva volcanics date from 4.53 to 3.62 Ma [38] and the island is composed of the outer remnant of a collapsed shield volcano and, within its crater, a younger, smaller post-shield eruptive formation.

The local climate is mesic tropical with air temperatures averaging between 25° and 27°C [39, 40]. Much of the archipelago’s rainfall derives from eastern trade winds. Variation in annual rainfall is not particularly marked, but the period from May to September is relatively wet and from October to April is comparatively dry, with an annual range between 700 and 1500 mm. Inter-annual rainfall, in contrast, can be quite variable, with both multi-year droughts and periods of extreme rainfall [40].

The archipelago has long been recognised for its biodiversity, with consequential radiations in birds, arthropods, snails, flowering plants, and even marine organisms [37, 41–43]. A variety of habitats are afforded by the rugged topography and geographic variation in rainfall. The contemporary indigenous vegetation includes montane cloud forests, moist-to-wet rainforests, and mesic-to-dry forests [37, 44, 45]. The indigenous vascular flora at present is comprised of approximately 333 taxa, of which 48% are endemic [37]. Another 33 to 37 plants are considered Polynesian introductions [20, 46, 47], some of which have only recently been archaeologically demonstrated [36, 48]. Over the period of human settlement, the indigenous flora has largely disappeared from the Marquesan lowlands, having been replaced in prehistory by productive arboricultural systems centred on *Artocarpus altilis* (breadfruit) [36, 49, 50]. More recently fast-growing, exotic grasses, shrubs, and trees have outcompeted many indigenous species [37, 51]. Grazing ungulates (goats and sheep), introduced early in the post-European period, have “completely denuded” some areas [52, p. 25], especially in drier localities [7, 53, 54]. Consequently, current biotic distributions and associations have been greatly altered relative to those observed early in the historic contact period, and conditions today are considerably different from those encountered by the original Polynesian colonists.

The exceptional Marquesan arthropod fauna also is of interest here. According to Ramage [55] this includes 1198 species, of which 681 are considered endemic to the Marquesas (53%), 138 introduced (11.5%), and the remainder mostly identified as indigenous, with some taxa of uncertain status. The majority of the endemic species derive from five orders: Coleoptera (beetles), Diptera (flies), Hemiptera (true bugs), Hymenoptera (wasps, bees and ants) and Lepidoptera (butterflies and moths). All of these contain radiations in at least two genera, with more than 10 species (and up to 76 species) restricted to the Marquesas, many being single island endemics. Important collections of terrestrial arthropods were made across the region by the Pacific Entomological Survey during the late 1920s and early 1930s, with numerous individual papers on Marquesan insects ultimately bound into three volumes that were published as Bishop Museum Bulletins in 1932, 1935 and 1939 [56–58]. Adamson [59] summarises the collecting activities on Nuku Hiva, noting that the most intensive collections were made on high elevation ridges where the greatest diversity of insects were found, with many fewer taxa at lower elevations. More recent research has focussed primarily on targeted collection of specimens for revisionary and molecular research into a relatively small component of the fauna, as summarised in Roderick and Gillespie [60]. The relationships of the arthropod fauna, where known, are like those of the closest regional archipelagos, including the Society, Hawaiian, and Austral Islands, and to a lesser extent the southern Cook Islands, Tuamotus and Sāmoa [43].

The role that humans have played in the development of the arthropod biota is perhaps best demonstrated by the ant fauna, which was recently reviewed by Ramage [61]. Most species of ants in the region are considered historical introductions; however, these interpretations are based exclusively on post-European contact data. The recent fossil record demonstrates that the assembly of the ant communities of eastern Polynesia is much more complex than the post-contact historical data suggests [19]. These complexities include species present prehistorically that are not currently recorded in the fauna (due to extinctions) and the Polynesian introduction of a range of species that, in the absence of long-term records, are typically considered historical introductions.

### The study locality

Ho‘oumi Valley, the location of our study site, is a long narrow catchment, which extends from sea level to a maximum of 450 m, on the relatively wet, eastern coast of Nuku Hiva. It parallels the very large valley of Taipivai but is separated from this catchment by a steep and rugged ridge of ca. 200 m elevation. A small, permanent, and generally slow-moving river courses through Ho‘oumi, ultimately emptying into the sea. Although it largely runs northwest to southeast, as it nears the coast the river is directed southward by an accretionary beach ridge (Fig 2). The current geomorphic configuration contrasts with that depicted on an archaeological field map from the late 1950s [62], where the river is shown discharging directly into the bay. This map also illustrates an “old stream bed” immediately north of the contemporary river channel, suggesting the channel has migrated southward over time. The current river course is probably also partly the result of purposeful channelization, with the main valley road running along the northern edge.

On either side of the river the land is fairly flat, the southwest side being low and muddy, and the northeast side slightly higher, more stable, and the location of a few traditional Marquesan dry stone masonry structures known locally as paepae [62, pp. 55–7, 63]). During the late Holocene sea highstand described for many central East Polynesian islands [64, 65] the low-lying area to the southwest may have been a tidal marsh. Today the Ho‘oumi shoreline is fringed by a narrow white-sand beach that indicates an offshore reef. This is also suggested by massive wave-cast coral heads that geomorphologists attribute to the 1946 Aleutian-generated tsunami [66–68].

Archaeological excavations in the late 1950s by Robert Suggs [62] uncovered evidence of two prehistoric cultural occupation layers at Ho‘oumi Beach, separated by a thick deposit of sand that may represent a palaeotsunami [63]. The upper cultural stratum is contemporaneous with the traditional raised stone house foundations (*paepae*) that persist on the northern coastal flat, while the lower cultural layer was associated with simple flagstone style pavements. In 2011, new archaeological research was carried out [63] to acquire chronological and palaeoenvironmental context for Suggs’ [62] earlier findings, which have figured importantly in archaeological reconstructions of traditional Marquesan history. The excavations were restricted to an area approximately 50 by 80 m on the northeast side of the river and to land under government jurisdiction, specifically within 50 m of the coast. As part of this initiative, the two cultural occupations were ^14^C-dated, the upper layer to the late prehistoric period and the lower occupation to between the 13^th^ and 14^th^ centuries AD [63]. Notably, both occupations are stratigraphically superior to, and radiometrically post-date, the exceptional anaerobic deposit reported here.

## Materials and methods

### Field collections

The study area was located ca. 70 m north of the contemporary Ho‘oumi River channel, on dry and somewhat elevated ground. The upper three strata of Trench 1 were unremarkable and generally lacking in cultural materials. Layer IV, in contrast, was unusually rich in well-preserved organic materials. Large blocks of compact, intact sediment were taken from five successive depth intervals within this layer and the exposed surfaces of each sample trimmed in the field to remove potential contaminants.

All necessary permits were obtained for the described study to comply with relevant regulations. The field study was carried out under Gouvernement de la Polynésie française, Ministère de la Culture et de l’Artisanat permit No. 1079, with authorisation from the Délégation à la recherche (de la Polynésie française), and assistance from the Service de la Culture et du Patrimoine. Field authorization was predicated on advance permissions from local government officials on Nuku Hiva for the archaeological study, specifically the Mayors of Hatiheu and Taiohae. Consultation was also carried out with local community members on the island in advance of the field study. Given that no human subjects, human remains, or living animals were involved in this study, no ethics approval was required by The University of Auckland Human Participants Ethics Committee. Archaeological samples, including the sediment samples analysed for this study, were exported to New Zealand with authority from the French Polynesian government Service de la Culture et du Patrimoine. Additional information regarding the ethical, cultural, and scientific considerations specific to inclusivity in global research is included in the S1 File.

### Radiocarbon dating

AMS dating was carried out on short-lived materials (SLM), specifically fruiting structures that develop and mature within a few years [69–71], with one exception. The latter was a small, culturally-modified timber where the age of the artefact was of particular interest. None of the AMS samples were carbonized. The conventional radiocarbon ages were calibrated using OxCal 4.4.4 [72] and the SHCal20 curve [73].

Two Layer IV samples were initially processed by Beta Analytic Testing Laboratory (Florida, USA). To evaluate the stratigraphic integrity of Layer IV, another two samples were submitted to the Waikato Radiocarbon Dating Laboratory (WRDL) (Hamilton, New Zealand). As these results were at odds with the initial Beta Analytic results, additional samples were submitted for analysis, including one from the overlying Layer III. One sample (Wk-49524) was also run twice by WRDL as a cross-check for contaminants. Additionally, splits of two samples initially run by WRDL were re-run by the University of California Irvine W. M. Keck Carbon Cycle Accelerator Facility in a blind test where they carried out pretreatment and graphitisation.

### Microfossils: Pollen and phytoliths

To evaluate the potential for microfossil recovery, a single bulk sediment sample (Acc. 7246) was taken from the middle section of Layer IV and submitted to commercial analyst Mark Horrocks of Microfossil Research, Ltd. for a pilot analysis of pollen, phytolith, and starch. For the pollen analysis, a standard acetolysis method was employed [74]. A total of 150 pollen grains and spores were counted, and scans undertaken to identify types not found during the formal counting. Sub-samples were prepared for phytolith and starch analysis using a density separation method [75].

### Macrofossils: Subfossil plants and arthropods

Five subsamples of organic sediment, each weighing approximately 500 grams, were systematically processed for plant and arthropod remains. Samples were disaggregated in 5% tetrasodium pyrophosphate and the lighter organic sediment washed though nested sedimentological sieves (2 mm, 1 mm, 500 μm, with the finest being 250 μm), leaving a fraction of heavier mineral and calcareous bioclasts. This heavy inorganic fraction was dried and examined for plant and arthropod material. The resulting organic sieve fractions were sorted in water using a binocular microscope and all potentially identifiable plant and arthropod materials were removed and stored separately in 70% ethanol.

Plant macrofossils were sorted to morphotaxa, counted, and where possible identified by Lewis using a personal reference collection and published sources [76–84]. Arthropod material was identified by Porch using material from southeastern Polynesia in Porch’s research collection housed in the School of Life and Environmental Sciences, Deakin University, and the considerable literature on the arthropod fauna of the region. Mites were not identified beyond major group because the modern fauna of Nuku Hiva is almost completely unknown and the fauna of the Marquesas has not been extensively studied for almost 100 years. For both plant remains and arthropods, taxa that could not be definitively identified to family were given morpho-taxon identifiers and are included in the results. Plant and arthropod specimens recovered from the Ho‘oumi bulk samples are housed at the Deakin University (Australia), School of Life and Environmental Sciences Palaeo-Laboratories. Additional sediment samples and macroplant remains obtained in excavation are housed at the University of Auckland (New Zealand), School of Social Sciences, Roger C. Green Archaeological Laboratory.

## Results

### Stratigraphic sequence

**Fig 3:**
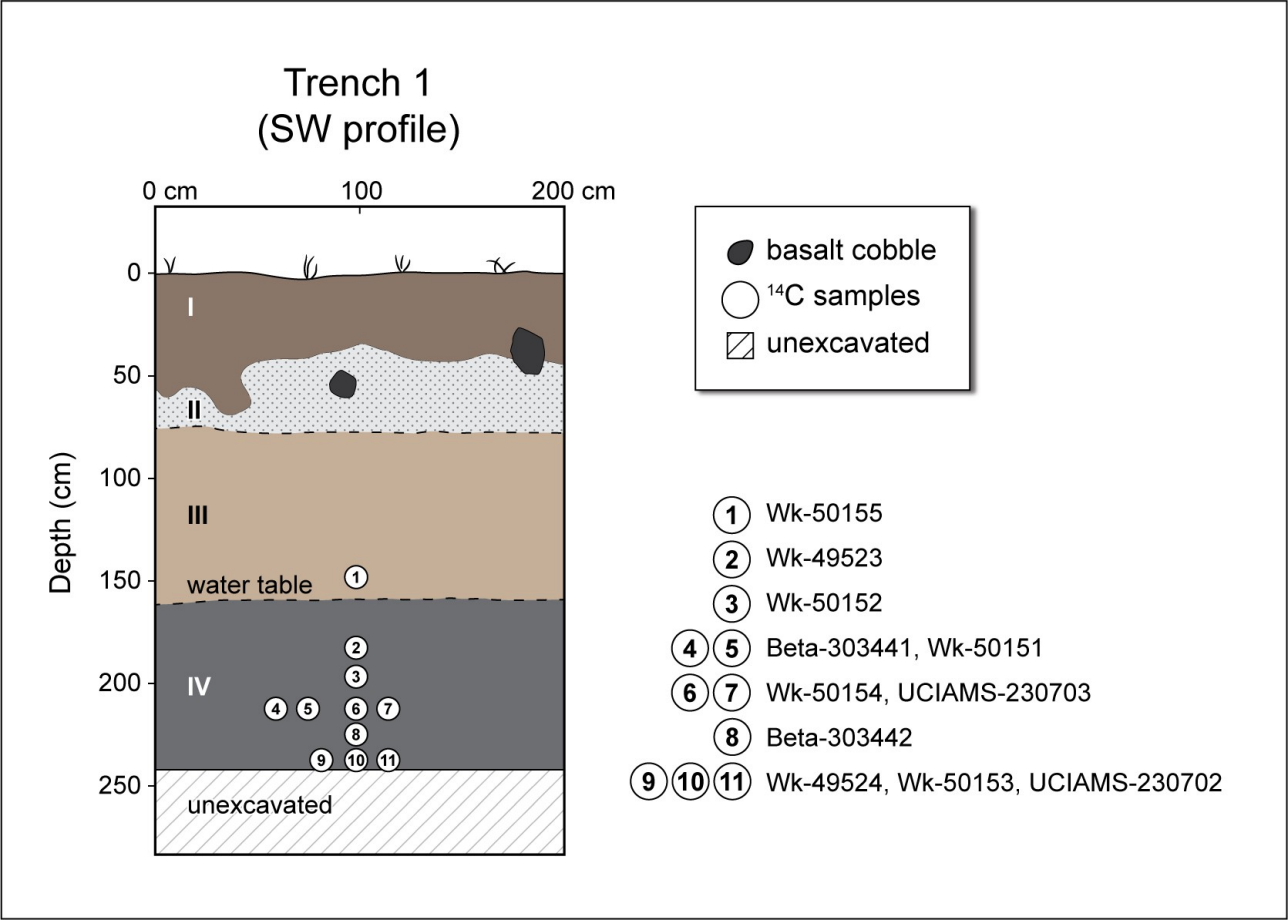
Stratigraphic profile of Trench 1, Ho‘oumi Beach, showing the location of the eleven radiocarbon samples.

Four stratigraphic layers were observed in excavation, largely differentiated on colour as all were of sandy textures (Fig 3, Table 1). No evidence of *in situ* cultural activities was observed within Trench 1, contrasting with findings from three other trenches to the north, situated on slightly higher ground [63]. Layer II of Trench 1 may represent a palaeotsunami deposit based on evidence from other nearby trenches. The Layer III/IV boundary in Trench 1 was marked by the top of the water table. Layer IV was rich in organic matter, including both micro- and macro-botanical remains, well preserved arthropods, and variable concentrations of microcharcoal. Excavations extended to 250 cm below the current ground surface but did not reach bedrock, again contrasting with findings from other trenches to the north where coralline bedrock was encountered at 160 and 200 cm below surface [63].

**Table 1:** Characteristics of Trench 1 strata, Ho‘oumi Beach.

Layer	Depth (cmbs)	Munsell colour	Soil texture	Soil structure	Consistence & plasticity	Clastics	Lower boundary	Primary depositional agent
**I**	0–40	Dark gray (10YR 4/1, dry)	Loamy medium to fine sand (marine sand ca. 5–15%)	Moderate, coarse crumb	Dry, slightly hard	Small (<1cm) charcoal fragments	Abrupt, irregular	Fluvial
**II**	ca. 40–75	Light brown-gray (10YR 4/2, dry)	Loamy very coarse sand (marine sand ca. 80%)	Weak, medium crumb	Dry, soft	Fine charcoal and occasional land snail	Abrupt, smooth	Fluvial: mainly marine
**III**	ca. 75–155	Dark gray-brown (10YR 4/3, dry), light brown-gray (10YR/6/2, dry)	Loamy coarse sand (marine sand ca. 40–50%)	Moderate, coarse to very coarse crumb to granular	Dry, slightly hard		Abrupt, smooth	Fluvial: mixed, terrigenous dominant
**IV**	155–250+	Black (10YR 2/1, wet)	Silty sand (mixed sand ca. 55%)	Structureless, massive	Wet, sticky, slightly plastic	Well preserved botanical remains and micro-charcoal	Not visible	Low energy fluvial: mainly terrigenous

Grain size analysis of the sediment fines (≤2 mm) from Layer IV was conducted using a laser granulometer. The results indicate the fines were “very poorly sorted” silty sand, following Folk [85]. The prevalence of very fine to fine sand, with considerable silt and some clay, indicates generally low energy, shallow water conditions, dominated by riverine influences but possibly with some occasional low energy marine deposition. This reflects the ecotonal location of the trench, just under 70 m from the bank of what is now a small, slow-moving, channelised river and ca. 50 m from the contemporary shoreline of a deep, generally protected, embayment (Fig 2). Outside of the varied organic remains, larger clastics were generally lacking, suggesting that the former accumulated *in situ*. Microcharcoal particles in the Layer IV samples point to burning at some distance from the study site, most likely upstream, and possibly in conjunction with anthropogenic forest clearance; macrocharcoal remains were lacking. The overlying sediments of Layer III mark a turning point in the local depositional regime. Subsequent inundation of the area by a palaeotsunami or storm surge (Layer II) probably contributed to subsequent shoreline aggradation, the evolving beach ridge, and shifts in the river course and outflow.

### Radiocarbon dating

**Fig 4:**
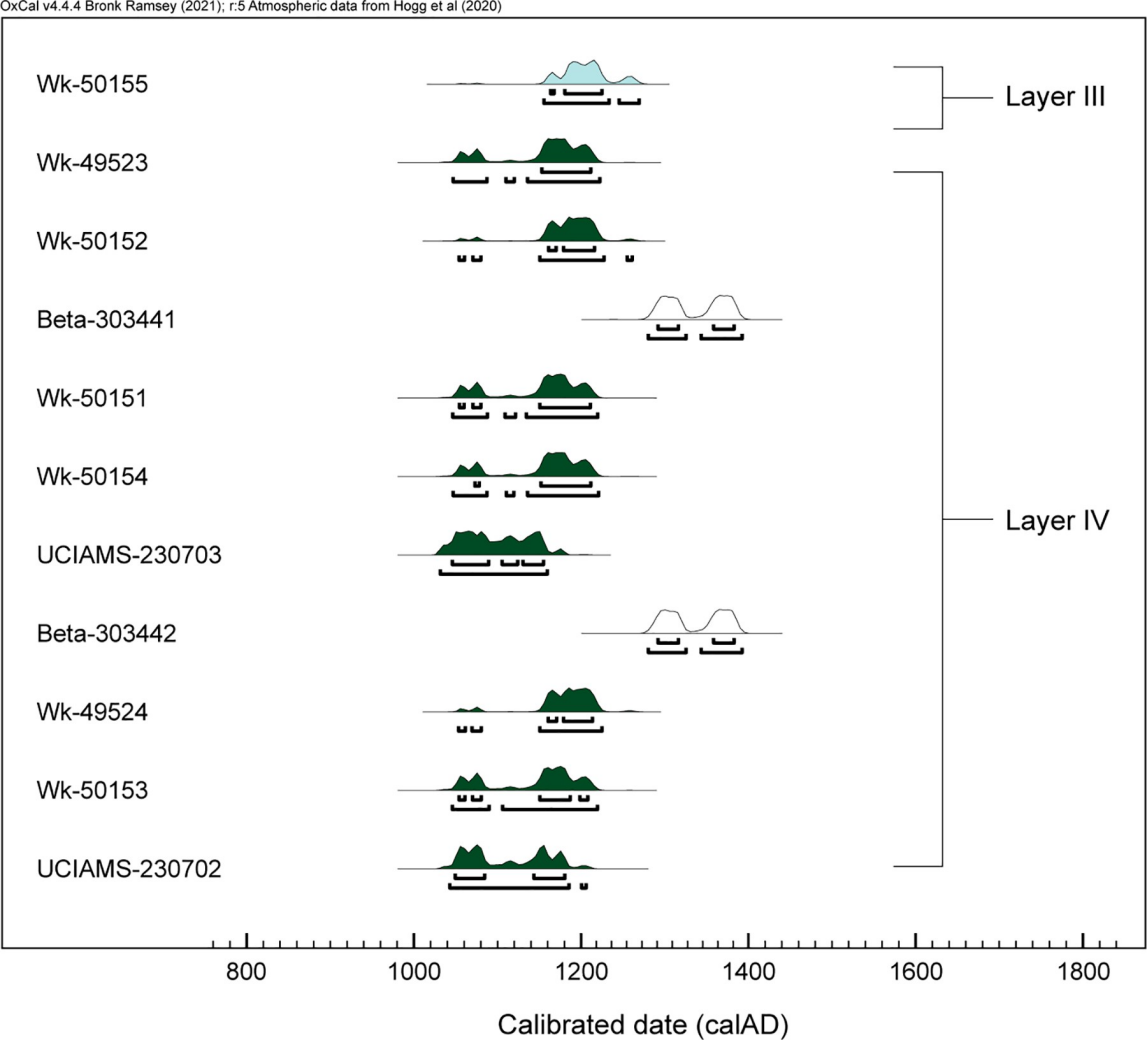
Oxcal plot of calibrated AMS results from Layer IV, Trench 1, Ho‘oumi Beach (see Table 2 and text for sample details).

Eleven radiocarbon analyses (Table 2, Fig 4) provide chronological context for human activities in this catchment and for the botanical and arthropod records presented below. The series of ^14^C samples was analysed incrementally, in an effort to answer specific questions about the Layer IV deposit and inter-lab variability in dating results.

**Table 2:** Trench 1, Ho‘oumi Beach (Nuku Hiva Island) radiocarbon sample details, analytic results, and calibrations^a^.

^14^C Lab Number	Acc. No.	Provenience^b^	Material	Type of analysis	d^14^C^0^/_00_	Conventional ^14^C age BP	Cal AD 95.4%	Cal AD 68.2%	Comments
**Wk-50155**	6731	Layer III	*Pandanus* fruit key	AMS	Na	886 ± 25	1155–1269	1163–1225	Layer above IV
**Wk-49523**	7243	Layer IV, bulk sample 1, ca. 175–190 cmbs (cm below surface)	*Pandanus *fruit key	AMS	Na	928 ± 26	1047–1222	1153–1211	Dates top of IV
**Wk-50152**	7244	Layer IV, bulk sample 2, ca. 190–205 cmbs	*Cocos nucifera* immature fruit	AMS	Na	905 ± 25	1054–1261	1161–1216	Dates upper part of IV
**Beta-303441**	7066	Layer IV, ca. 190–235 cmbs	Adzed *Sideroxylon* sp. timber	AMS	-25.6	710 ± 30	1280–1392	1292–1383	Dates artefact & middle of IV; from field sample bag 7065 but subsequently given a unique number.
**Wk-50151**	7066	Layer IV, ca. 190–235 cmbs	Adzed *Sideroxylon* sp. timber	AMS	Na	933 ± 25	1046–1220	1054–1211	Replicate of Beta-303441; dates artefact & middle of IV
**Wk-50154**	7245	Layer IV, bulk sample 3, ca. 205–220 cmbs	*Cocos nucifera* immature fruit	AMS	Na	930 ± 25	1047–1221	1073–1211	Dates middle of IV
**UCIAMS-230703**	7245	Layer IV, bulk sample 3, ca. 205–220 cmbs	*Cocos nucifera* immature fruit	AMS	-114.5 ± 2.1	975 ± 20	1031–1159	1046–1155	Replicate (split) of Wk-50154
**Beta-303442**	7061	Layer IV, bulk sample 4, ca. 220–235 cmbs	*Cocos nucifera* endocarp	AMS	-25.4	710 ± 30	1280–1392	1292–1383	Dates IV; lab queried tannin staining
**Wk-49524**	7247	Layer IV, bulk sample 5; ca. 235–250 cmbs	*Cocos nucifera *endocarp	AMS	Na	909 ± 24	1053–1225	1160–1213	Dates bottom of IV; also rerun by Petchey to check for contaminants (see text)
**Wk-50153**	7247.2	Layer IV, bulk sample 5; ca. 235–250 cmbs	*Pandanus* fruit key	AMS	Na	936 ± 25	1046–1219	1054–1208	Run as a check on Wk-49524.
**UCIAMS-230702**	7247.2 (^14^C sample split)	Layer IV, bulk sample 5; ca. 235–250 cmbs	*Pandanus* fruit key	AMS	-111.8 ± 2.1	955 ± 20	1043–1206	1049–1180	Replicate analysis (split) of Wk-50153

^a^ Calibrated using OxCal 4.4.4 [72] and SHCal 20 [73].

^b^ In the field, Layer IV was provisionally considered a subunit of Layer III and accordingly labelled IIIb. Following sedimentological and palaeobiological analyses, the decision was made to assign the stratum its current unique identifier. As a matter of record, IIIb was used in some of the original field notes.

Two range finder samples analysed in 2011 by Beta Analytic returned mid-13^th^ to late 14^th^ century age estimates (Beta-303441 and Beta-303442). In an effort to establish the onset and cessation of deposition within Layer IV, another two samples were submitted to Waikato Radiocarbon Dating Lab (WRDL) in 2019, one from the top (Wk-49523) and a second from the base of Layer IV (Wk-49524). These samples both returned mid-11^th^ to early 13^th^ century age ranges (95.4% prob.), overlapping with one another but at odds with the prior Beta Analytic results.

Subsequently, additional samples were submitted for ^14^C analysis. A sample from the overlying Layer III (Wk-50155) was intended as a constraint on the Layer IV results. This produced an age range that overlapped with, but was slightly later than, that from the top of Layer IV (Wk-49523). In an effort to understand the differing results from the two laboratories, a second sample (Wk-50151) was taken from the same adzed timber that provided Beta-303441. The result was consistent with the other WRDL analyses but offset from the two Beta Analytic results.

As a cross-check for contaminants, Sample Wk-49524 was re-run by WRDL; it returned a CRA (879 ± 24) that was quite similar to the original WRDL result (909 ± 24) reported in Table 2. To further evaluate Wk-49524, another sample was submitted from the base of Layer IV (Wk-50153); again, the results were internally consistent, but offset from the Beta Analytic results. Finally, splits of two samples initially analysed by WRDL were processed by John Southon of the University of California, Irvine W. M. Keck Carbon Cycle Accelerator Facility (UCIAMS-230702 and -230703). The UCIAMS results were internally consistent and nearly identical to those provided by the WRDL.

In sum, results from nine samples, taken from the top, middle, and bottom of Layer IV, and in two cases on splits of the same specimens, are statistically indistinguishable at two sigma; these were analysed by WRDL and the University of California, Irvine AMS facility. These results contrast with the two Beta Analytic results and the lab offset is of some consequence. Potential causes are differences in modern or background standards or differences in pretreatments (A. Hogg, pers. comm, 2020). As inter-laboratory variation is increasingly under scrutiny, WRDL recently participated in a global comparison of radiocarbon laboratory performance and no consequential offsets were detected in that comparative exercise [86, A. Hogg, pers. com., 2020].

The approach taken here is consistent with advice on archaeological ^14^C “best practice” [70, 71]. Multiple AMS samples were run and these derived from three different kinds of short-lived materials (*Pandanus* keys, immature *Cocos* fruits, and mature *Cocos* endocarp) with the aim of excluding the possibility of in-built age. Sample Wk-50151, although from a timber, was derived from the exterior surface and the result overlaps with those on short-lived materials. The series as a whole provides a robust age estimate for the deposition of Layer IV, the larger assemblage of subfossil floral and faunal materials, and the subfossil arthropods.

**Fig 5:**
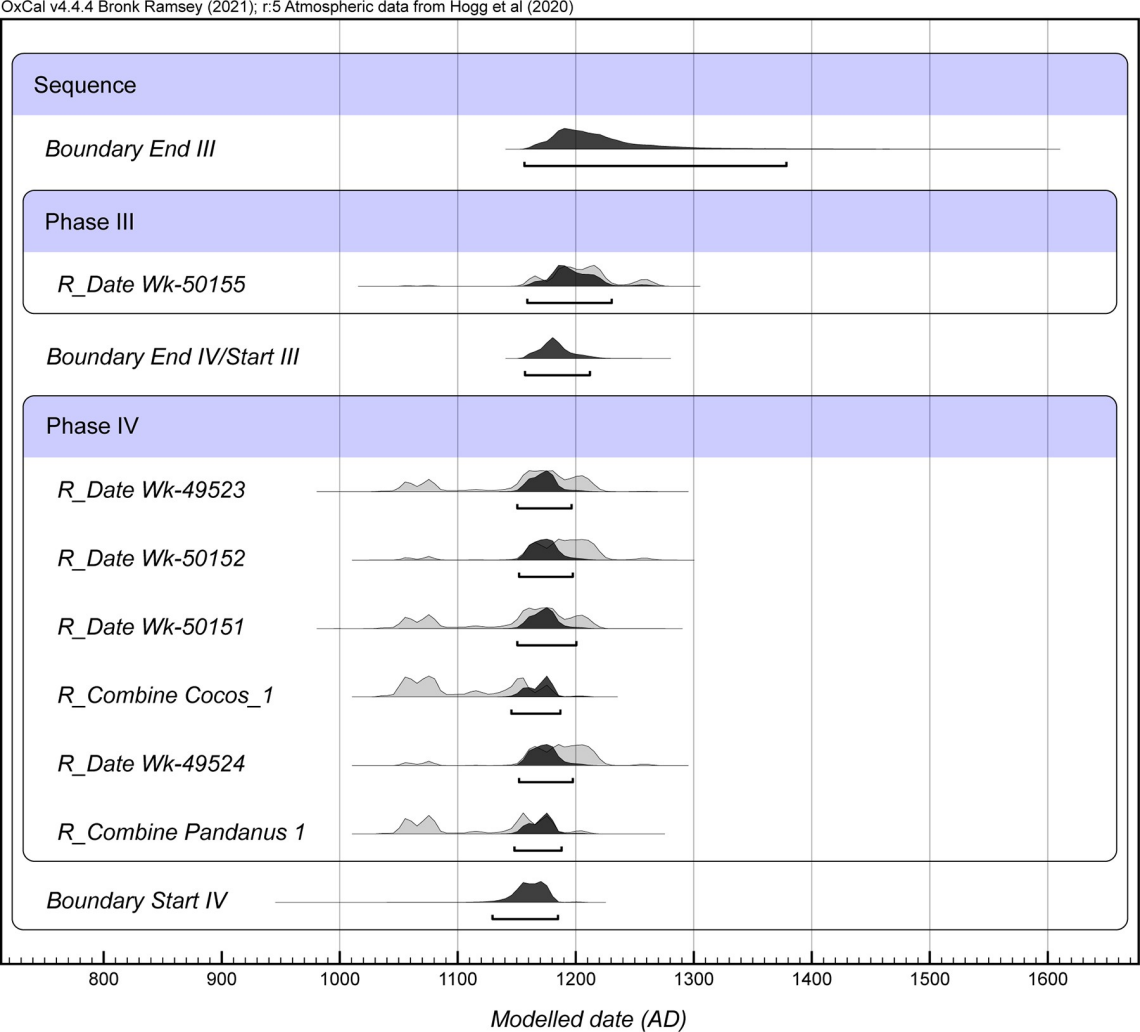
Bayesian age model for radiocarbon dates from Layers IV and III of Trench 1, Ho‘oumi Beach.

To further assess the ^14^C results (other than the two Beta outliers discussed above), we used the Bayesian sequence analysis of OxCal ver. 4.4.4 and the Outlier Model with the Outlier command applied to the *Sideroxylon* timber sample Wk-50151 (Fig 5; Bayesian model code in S1 Text). Results from specimen splits (Wk-50153 and UCIAMS-230702, *Pandanus* key; Wk 50154 and UCIAMS-230703, *Cocos nucifera* immature fruit) were combined. The model indicates that the most likely time interval (95.4% highest posterior density or HPD) for deposition of Layer IV lies in the mid-12^th^ century AD. Results show a Layer IV start estimate between *1129–1185 cal*. *AD* (95.4%) and an end boundary of *1157–1212 cal*. *AD*. At 95.4% HPD, the modelled Bayesian age for Layer IV lies between *1129 and 1212 cal*. *AD*. The Bayesian results also suggest that Layer IV accumulated quite rapidly. The single sample from Layer III indicates this stratum most likely accumulated between *1159–1269 cal*. *AD* (95.4%).

### Pollen and phytolith records

**Fig 6:**
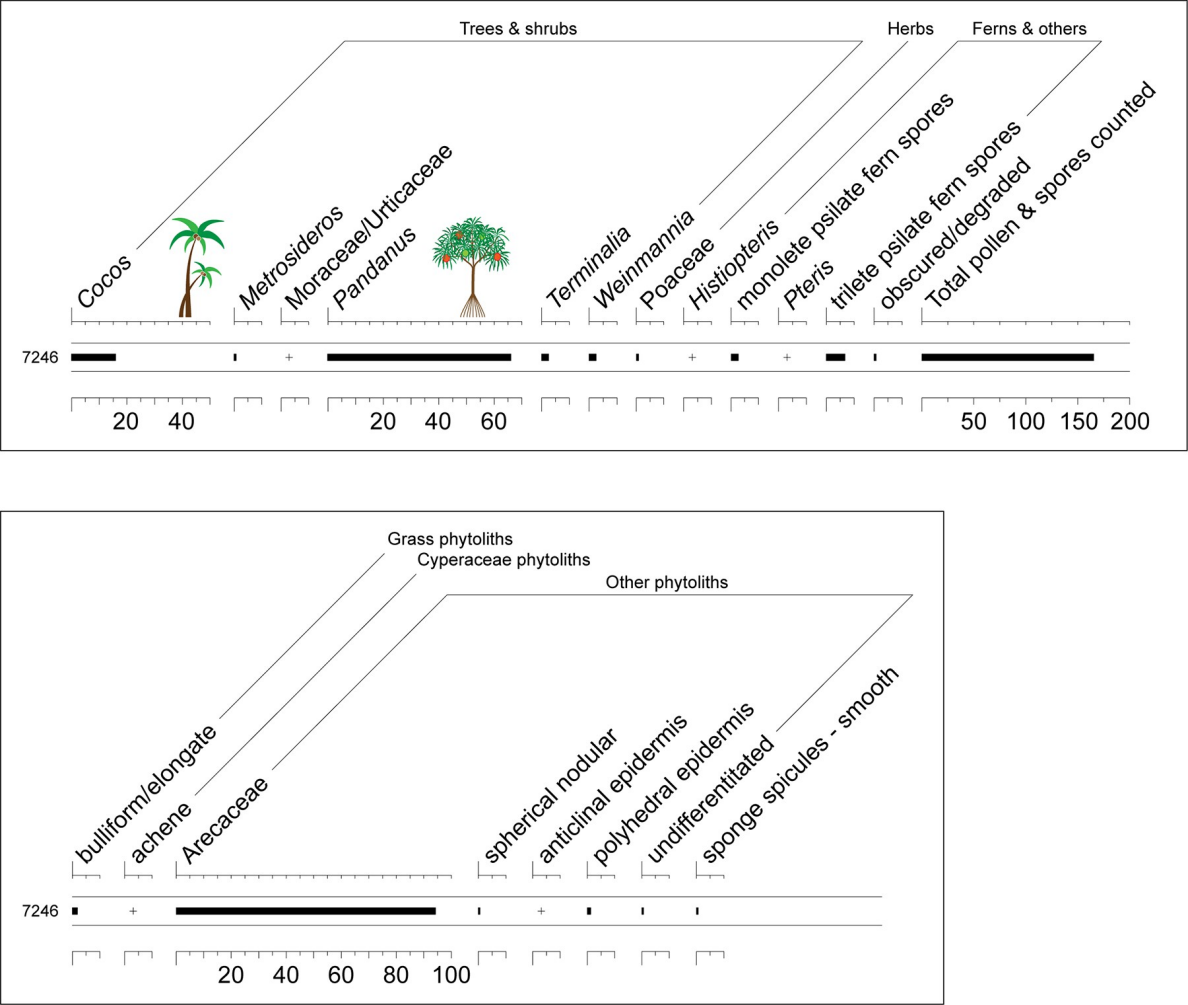
Microfossils assemblages from Layer IV, Ho‘oumi Beach. Top: Pollen record. Bottom: Phytolith and sponge spicule assemblage.

The microfossil sample from Layer IV provided a modest but informative record, although no starch microfossils were recovered [87]. The pollen sample was dominated by *Pandanus* (>65%), but *Cocos* (ca. 15%) was also moderately well represented (Fig 6, top). Native forest trees included *Pterophylla* (syn. *Weinmannia*, Cunoniaceae), *Metrosideros* (Myrtaceae), and *Terminalia* (Combretaceae). Several pollen grains were assigned to Moraceae/Urticaceae, a broad category that encompasses taxa of varied biogeographic origins. Marquesan members of the Moraceae family include the *Ficus prolixa* var. *prolixa*, *Streblus antropophagorum*, *Artocarpus altilis* (breadfruit), and *Broussonetia papyrifera* (paper mulberry)—the latter two being Polynesian introductions. Among the native Marquesan Urticaceae are two small trees/shrubs, *Pipturus* (three species) and *Boehmeria virgata*, as well as *Procris pedunculata*, a succulent herb that is common in wet valley forests and often epiphytic. Unfortunately, a definitive identification was not possible.

Other microfossils (phytoliths and sponge spicules) inform on the subcanopy vegetation, with both fern spores and grass pollen represented (Fig 6, bottom). *Histiopteris*, most likely *H*. *incisa*, is a fast-growing, sun-loving taxon that is a good indicator of natural or anthropogenically disturbed areas (e.g., landslides, treefall gap, fire, etc.). The phytolith assemblage, however, was dominated by Arecaceae (palm family), and only a small number of grass phytoliths were observed, suggesting that a closed canopy dominated the area. The sponge spicules were not further identified but may warrant future study as approximately one-third of the Marquesan sponges are endemics [88].

### Plant macrofossil assemblages

**Fig 7:**
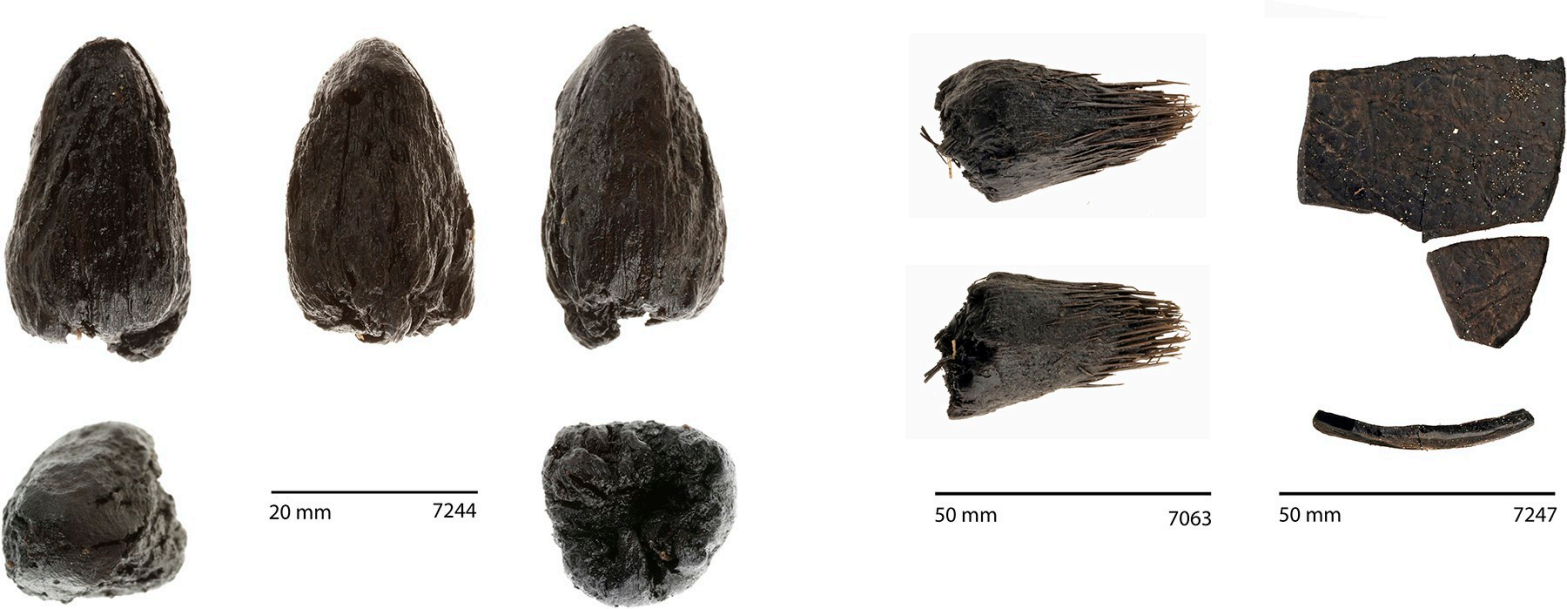
Examples of common macrobotanical materials. Left: immature *Cocos nucifera* fruit (five views); centre: *Pandanus* key (two views); right: *Cocos nucifera* endocarp (plan view and cross-section). (Photos by Tim Mackrell).

**Fig 8:**
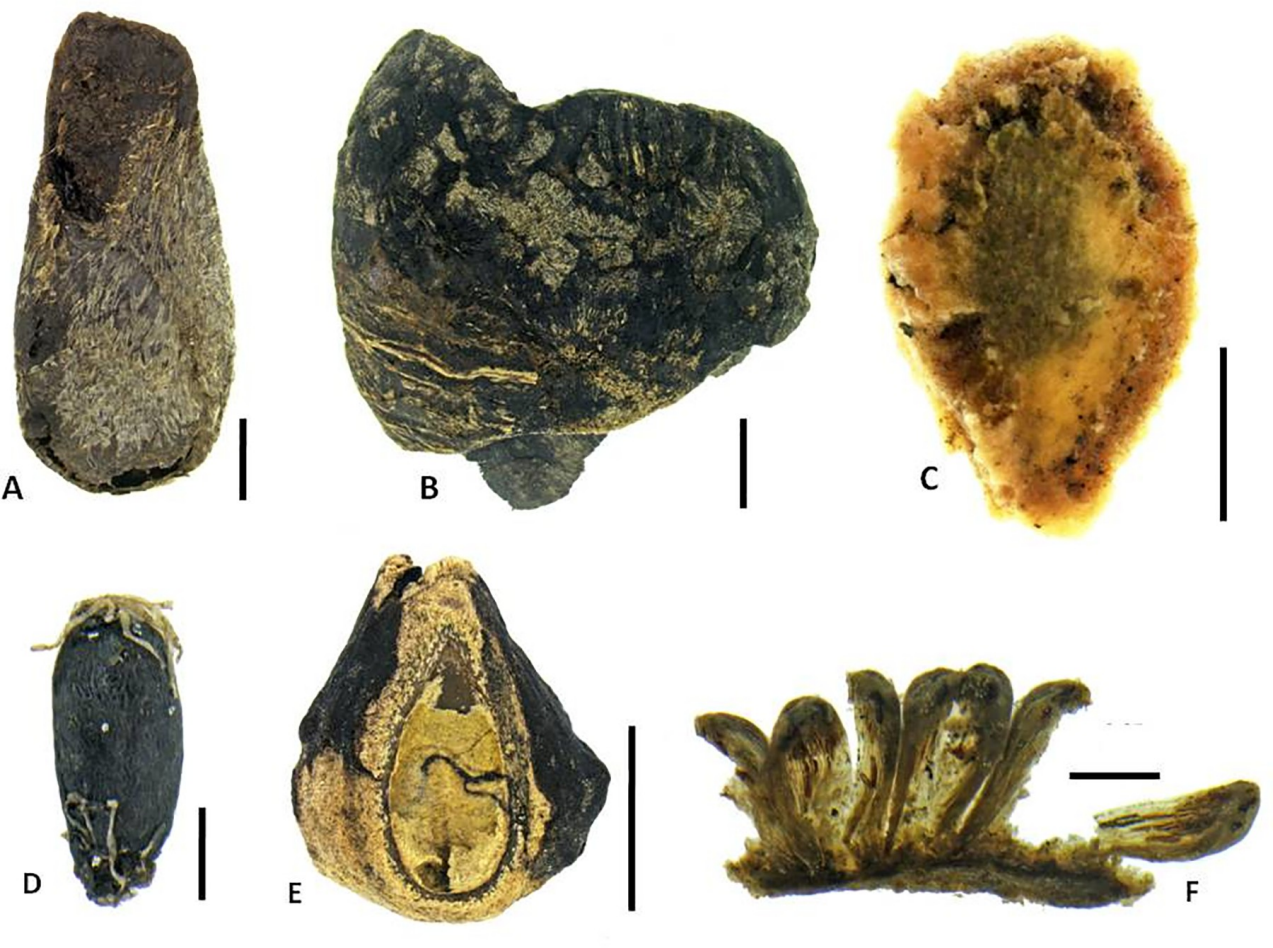
Representative plant macrofossils (seeds unless otherwise stated). A. *Morinda citrifolia*; B. *Pritchardia*-type (outer surface); C. Curcubitaceae indet.; D. *Pterophylla* cf. *marquesana*; E. *Premna serratifolia* (endocarp showing seed chamber); F. *Angiopteris marchionica* (sorus with sporangia). Scale bars: A, B, C, E, 2 mm; D, F, 0.25 mm. (Photos by N. Porch).

**Fig 9:**
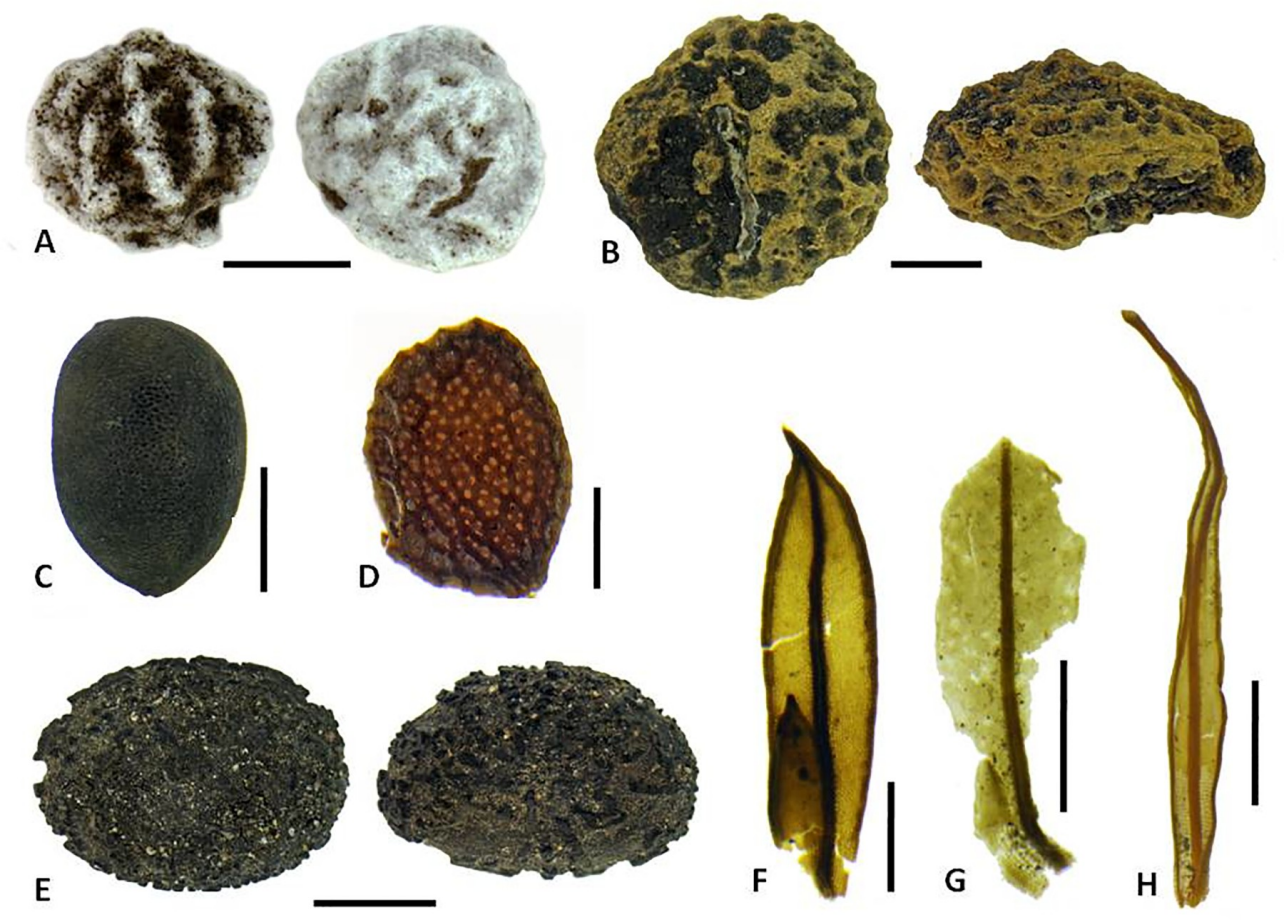
Plant macrofossils of potential extirpations or extinctions. Seeds: A. *Trema* cf. *orientalis*; B. cf. *Claoxylon* (ventral view; lateral view); C. *Acalypha*; D. *Mussaenda*; E. *Macaranga* (lateral view; dorsal view). Moss leaves: F. *Fissidens* cf. *raiatensis*; G. *Calymperes* cf. *moorei*; H. *Calympere*s cf. *tahitensis*. Scale bars: B, E, 2 mm; A, H, 1 mm; C, G, 0.5 mm; D, F, 0.25 mm. (Photos by N. Porch).

The plant macrofossil assemblage contains a diversity of seeds, fruits, thorns, anthers, fern sporangia and scales, and bryophyte leaves (some entire, but many fragmented). A total of 1768 plant macrofossil remains were identified; of these, 1716 are higher plants from 31 named taxa and 52 are bryophyte specimens. The assemblage is dominated by indigenous taxa (Tables 3 and 4) and various life forms are represented: trees, shrubs, herbs, gramminoids and ferns (Figs 7–9). Indications are that at least two vegetation communities are represented. Possible extinct taxa include *Mussaenda*, *Macaranga*, and *Acalypha*, none of which were previously known from the archipelago. In one case, *Mussaenda*, the subfossil specimens are morphometrically distinct from other species in the region (see below). While there is one modern endemic Nuku Hivan species of *Claoxylon*, its seeds are dissimilar from the subfossil [78]. As such, an extinct *Claoxylon* species may be present in the assemblage. The *Trema* seeds could derive from *T*. *orientalis*, which has only recently been recognised as indigenous [47].

**Table 3:** Summary of identified plant material (+ = present).

Family	Scientific name	English (Marquesan) name	Biogeographic status^a^	Macrofossil counts from five bulk sediment samples^b^					Micro-fossils^c^	Wood^d^	Contemporary vegetation zone; elevation^a^
				7243	7244	7245	7246	7247			
**TREES/PALMS**											
**Arecaceae**	*Cocos nucifera* L.	Coconut (*‘Ehi*)	Indigenous; prob. also Polynesian introduction				3		+	+	Sandy coasts where rainfall exceeds 75–100 cm per year
**Arecaceae**	*Pritchardia*-type	Fan palm	Indigenous	1				1			Rare [91]
**Asteraceae**	cf. *Oparanthus*	Unknown	Uncertain	35	7	22	60	9			Unknown
**Cannabaceae**	*Trema* cf. *orientalis* (L.) Blume	Unknown	Uncertain	2	2	1	2				Naturalised on Ua Pou, roadsides; 274–300 m; in other archipelagos, in riparian forest, secondary forest; 0–1200 m [79]
**Calophyllaceae**	*Calophyllum inophyllum* L.	(*Temanu*)	Indigenous	1						+	Coastal areas; 0–350 m
**Combretaceae**	*Terminalia* sp.	Poss. tropical almond (*Ma‘i‘i*)	Endemic or naturalized	1			1		+		*T*. *catappa* (naturalized) Coastal areas, also in coconut groves, and along roadsides; 0–380 m; *T*. *glabrata* var. *brownii* (endemic) Lowlands and around villages, except on Mohotani, where it occurs at higher elevations; 1–500 m
**Cunoiaceae**	*Pterophylla* (syn. *Weinmannia*) cf. *marquesana* (F.Br.) Pillon & H.C.Hopkins	Unknown	Endemic		1	2	5	1			Mesic to wet shrublands and forests, fern-lands, and cloud forests on crests and summits; 400–1200 m
**Euphorbiaceae**	cf. *Claoxylon*	Unknown	Uncertain		1						Unknown
**Fabaceae**	*Erythrina variegata* L.	Indian coral tree (*Kenae*)	Indigenous [23]							+	Steep, dry locations in secondary vegetation or mesic forests; 1–550 m
**Lecythidaceae**	cf. *Barringtonia asiatica* (L.) Kurz	Fish poison tree (*Hutu*)	Indigenous							+	Coastal vegetation and mid-elevation mesic forests, sometimes cultivated in villages; 0–370 m
**Malvaceae**	*Talipariti tiliaceum* (syn. *Hibiscus tiliaceus*) (L.) Fryxell	Possibly. Beach hibiscus (*Hau*) but could be *Hibiscus rosa-sinensis*	prob. Indigenous							+	Along streams, in seeps, and on moist slopes; 0–1000 m
**Malvaceae**	*Thespesia populnea* (L.) Sol. ex Corrêa	Pacific rosewood (*Mi‘o*)	Indigenous							+	Coastal vegetation and lowland mesic forest; 0–300 m
**Moraceae**	*Ficus* cf. *prolixa* G.Forst.^e^	Pacific banyan (*Aoa*)	Indigenous	132	95	94	432	178			In ravines, on steep slopes, and rocky outcrops; 10–1000 m
**Moraceae/ Urticaceae**	uncertain	Mulberry or Nettle family	Indigenous						+ ^f^		Uncertain
**Myrtaceae**	*Metrosideros collina* (Forst.) A.Gray	(*Heua*)							+		Mesic and wet forests, cloud forests, wet summit shrublands, and fern-lands; 300–1300 m
**Pandanaceae**	*Pandanus tectorius* Parkinson ex Du Roi	Screwpine (*Ha‘a*)	Indigenous		3	1			+	+	Mixed thickets, cultivated forest remnants and mesophytic rain forest; 5–850 m [46]
**Rubiaceae**	*Guettarda speciosa* L.	Beach gardenia (*Hano*)	Indigenous					1			Coastal forests, dry to mesic forests and savannah vegetation, often in valleys; 0–1000 m
**Sapotaceae**	*Sideroxylon* sp.	Unknown	Indigenous							+	Extinct: see [23]
**Verbenaceae**	*Premna serratifolia* L.	(*Va‘ova‘o*)	Indigenous		1						Coastal areas and dry forests to montane mesic and wet forests and shrublands; 2–960 m
**SHRUBS**											
**Euphorbiaceae**	*Acalypha* sp.	Unknown	Uncertain	7	12	3	3	2			Unknown
**Euphorbiaceae**	*Macaranga*-type	Unknown	Uncertain				1	1			Unknown
**Gesneriaceae**	*Cyrtandra* cf. *thibaultii* Fosberg & Sachet	Unknown	Endemic	1		2	2	1			Mesic to wet forests and shrublands; 700–1170 m. Other Nuku Hiva *Cyrtandra* also occur above 700 m, in wet forests, cloud forests and woodlands
**Loganiaceae**	*Geniostoma* cf. *hallei* Fosberg & Sachet	Unknown	Endemic				1				Montane wet forests and shrublands on steep upper slopes and summits in cloud zone; 620–1100 m
**Lythraceae**	cf. *Pemphis acidula* J.R.Forst. & G.Forst.	Unknown	Uncertain				1				Coastal areas
**Moraceae**	*Pipturus* cf. *argenteus* (G.Forst.) Wedd.	(*Hoka*)	Indigenous	111	113	47	132	75			Plantations, roadsides, and secondary forests to native dry, mesic, and wet forests, often in clearings or along streams; 0–1010 m
**Phyllanthaceae**	*Phyllanthus pacificus*-type	Unknown	Uncertain		1	1	1				Mesic and wet forests and shrublands; 25–1200 m
**Rubiaceae**	*Morinda citrifolia* L.	Indian mulberry (*Noni*)	Indigenous	1							Secondary and native, dry to wet shrublands and forests; 0–700 m
**Rubiaceae**	*Mussaenda* sp.	Unknown	Not previously recorded in the Marquesas	1	1			2			unknown
**Solanaceae**	*Solanum* cf. *repandum* G.Forst.	Unknown	Indigenous	2	1		3				Around villages; 0–100 m
**HERBS**											
**Cucurbitaceae**	uncertain	Gourd family	Uncertain		1			1			
**Cyperaceae**	uncertain	Sedge family	Uncertain	5	10	6	6	3			
**Cyperaceae**	*Fimbristylis* sp.	Unknown	Native	1							
**Portulacaceae**	*Portulaca* sp.	Unknown	Uncertain	6	11	2	3	2			
**Poaceae**	uncertain	Grass family	Uncertain		1						
**Solanaceae**	*Nicotiana* sp.	Unknown	Uncertain	5	2		4	1			
**FERNS**											
**Dennstaedtiaceae**	*Histiopteris* cf. *incisa* (Thunb.) J.Sm.	Unknown	Indigenous?						+		
**Pteridaceae**	*Pteris* sp.	Unknown	Uncertain						+		
**Marattiaceae**	*Angiopteris marchionica* E.D.Br.	Unknown	Endemic	2	1	1	13	2			Wet forests and shrubland, often in humid valleys and gulches; 200–1100 m
**Maratticeae**	*Ptisana salicina* (Sm.) Murdock	Unknown	Indigenous				5	1			Mesic forests, wet forests, cloud forests, and wet shrublands, often in upland ravines; 500–1100 m
			Macrofossil total	314	264	182	675	281	1716		
			No. of macrofossil taxa	17	18	12	18	16			

^a^ Based on https://naturalhistory2.si.edu/botany/marquesasflora/ [81] unless otherwise indicated.

^b^ Identified by Tara Lewis (this article).

^c^ Identified by Mark Horrocks [87].

^d^ Identified by Jennifer Huebert [in 63].

^e^ The presence of *Platyscapa* fig wasps provides corroborative evidence for this determination.

**Table 4:** Preliminary identifications and counts of subfossil bryophytes from five bulk sediment samples.

Family	Scientific name	Acc. 7243	Acc. 7244	Acc. 7245	Acc. 7246	Acc. 7247
**Liverworts**						
Lejeuneaceae	*Lopholejeunea*-type^a^	1			1	
Lejeuneaceae	Lejeuneaceae 2^a^	6	1			
**Mosses**						
Calymperaceae	*Calymperes* cf. *moorei* E.B.Bartram^b^		1	2		1
Calymperaceae	*Calymperes* cf. *aongstroemii* Besch.^b^		1	1	1	
Calymperaceae	*Calymperes* cf. *tahitense* Mitten^b^			4		
Calymperaceae	cf. *Syrrhopodon*^b^				1	
Fissidentaceae	*Fissidens* cf. *aoraiensis* H.O.Whittier & H.A.Miller^a^		4	6		1
Fissidentaceae	*Fissidens* cf. *raiatensis* E.B.Bartram^b^	2				
Poss. Neckeraceae	*Homaliodendron*-type^a^				1	
Poss. Neckeraceae	Neckeraceae-type ^a^ ^(7243);^ ^b^ ^(7246)^	2			2	
Uncertain	Bryophyte 4^a^	5			3	
Uncertain	Bryophyte 6^a^	1				4
	Total	17	7	13	9	6

^a^ Stem fragment with leaves.

^b^ Leaf.

Endemic forest elements include trees and shrubs such as *Cyrtandra* cf. *thibaultii*, *Pterophylla* cf. *marquesana* and *Geniostoma* cf. *hallei*. There are four endemic species of *Cyrtandra* on Nuku Hiva. Based on the similarity of size, sculpture and the line drawing of the seed of *Cyrtandra thibaultii* in Gillet (as *C*. *jonesii*) [figure 91 in 80], the subfossil was determined as *Cyrtandra* cf. *thibaultii*. There are two endemic species of *Geniostoma* on Nuku Hiva. The size of the subfossil suggests it is most likely *Geniostoma* cf. *hallei*, as its seeds are the larger of the two species [46]. Two relatively large ferns, *Ptisana salicina* and *Angiopteris marchionica*, are also present. These taxa are found today in mesic, moist, and wet montane forests [46, 47, 84] and are largely restricted to mountain ridges and slopes (700–1150 m) (Table 3). Their presence in the Ho‘oumi deposit may indicate that these forests elements once extended to lower elevations. Pioneer taxa such as *Pipturus* cf. *argenteus* and *Ficus* cf. *prolixa* are abundant, and less commonly *Morinda citrifolia* and *Trema* cf. *orientalis*, suggesting the possibility of disturbances within the associated forest communities. Although *Morinda citrifolia* has been considered a Polynesian introduction in the past, Porch and Prebble’s work elsewhere in the central East Polynesian region suggests it may pre-date human arrival.

Elements of lowland coastal forests are also present. *Guettarda speciosa*, *Premna serratifolia*, *Cocos nucifera*, *Pandanus tectorius* and *Calophyllum inophyllum* are among the recovered fruits and seeds (Fig 7), though the first two can also be found at altitude. Remains of *Pandanus* (fruit keys, trunk fragments, and leaves) and *Cocos* (immature fruits, fragments of mature endocarp, and wood) were particularly common, suggesting these taxa grew in the immediate area. Seeds of Cucurbitaceae (Fig 8) and Cyperaceae may derive from native taxa or could be Polynesian introductions; unfortunately they could not be identified to lower taxonomic levels with our current reference collections (see further discussion below). *Pritchardia*-type palm seeds (Fig 8), probably deriving from the lowland coastal forest, were present in two samples as fragments, but no complete specimens were recovered.

Preliminary bryophyte identifications indicate two Lejeuneaceae taxa (leafy liverworts), and two *Fissidens* species, three *Calymperes* species, one *Syrrhopodon* species, and two possible Neckeraceae taxa (mosses) (Table 4, Fig 9). *Fissidens* cf. *raiatensis*, *Fissidens* cf. *tahitensis* and *Calymperes* cf. *moorei* represent new records for the Marquesas Islands [82, 83], though more recent studies on the Marquesan bryophytes are lacking.

These seed and fruit assemblages are complemented by a modest collection of woody materials, previously reported in [63] and identified by Jennifer Huebert. Indigenous species, following Wagner and Lorence [81], that are represented only by wood include cf. *Barringtonia asiatica*, *Erythrina variegata*, *Talipariti tiliaceum* (syn. *Hibiscus tiliaceus*; or possibly *H*. *rosa-sinensis*), *Sideroxylon* sp., and *Thespesia populnea*, as well as rhizome/trunk fragments that are suggestive of Cyatheaceae. Notably, *Talipariti*/*Hibiscus* is not well represented in the Layer IV wood charcoal assemblage. This is consistent with patterns elsewhere on Nuku Hiva, where the taxon is best represented in mid- to late prehistoric charcoal assemblages [23], suggesting its distribution expanded in concert with the growth of anthropogenic environments.

**Fig 10:**
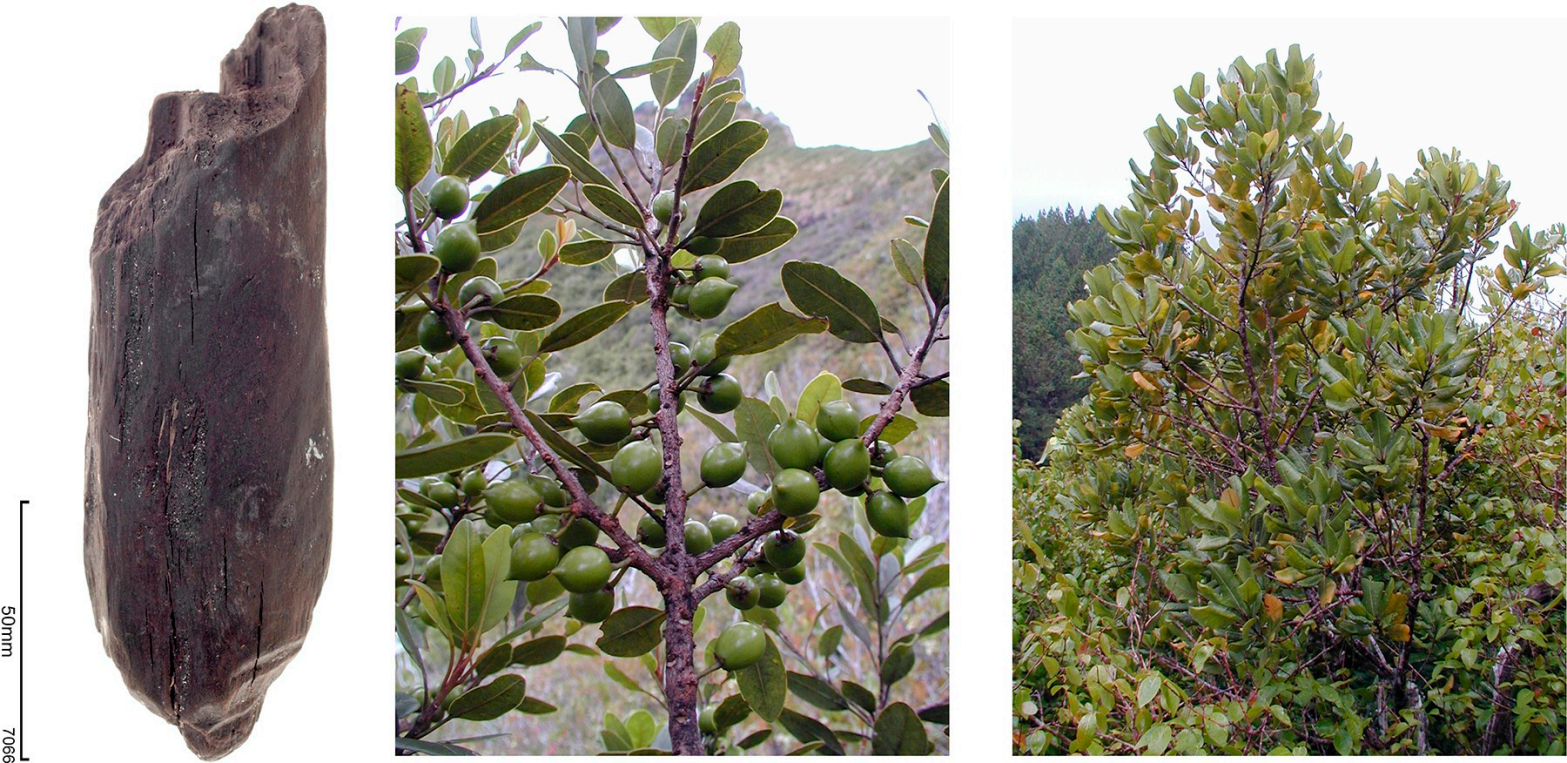
Left: archaeological specimen of *Sideroxylon* sp. from Layer IV, Trench 1; note cuts at lower end. (Photo by Tim Mackrell); Centre: *Sideroxylon polynesicum* in fruit, Mount Taga, Rapa Island, 2002. (Photo courtesy of Jean-Yves Meyer); Right: mature *Sideroxylon polynesicum*, Matotea, Raivavae Island, 2002. (Photo courtesy of Jean-Yves Meyer).

The most notable subfossil wood specimen is the adzed *Sideroxylon* (syn. *Nesoluma*) timber (Fig 10). Roughly 15 cm in diameter, one end of the specimen angles to a point, at a roughly 45 degree angle. The tapered end is marked by two to three narrow, obliquely angled steps. The modifications are consistent with those that result when a traditional Polynesian stone adze is used in an oblique chopping action on a standing small diameter tree and contrast with the jagged and asymmetrical breaks that arise from natural tree falls. The modifications are also consistent with stone adze marks found on more formal Polynesian artefacts. More speculatively, the tapering in the round of this small diameter timber might suggest secondary shaping for a particular use.

Deposits elsewhere on Nuku Hiva from later time intervals indicate that this endemic taxon was once a common component of lowland forests and the dense wood was often used as a firewood [23, 89]. The specimen illustrated here is most likely a remnant of forest clearing activities, but it could also have been felled for fuel or modified for use as a fence post. This genus of the Sapotaceae family, now extinct in the Marquesas, is known solely from archaeological materials.

Prior to our Marquesan studies, three *Sideroxylon* species were known for East Polynesia [90]. *S*. *polynesicum*, a shrub to medium tree, is the most widely distributed, being present in the southern Cook (Mangaia), Austral (Rapa and Raivavae), and Hawaiian Islands. Two endemic species are recognised: *S*. *nadeaudii* persists in the Society Islands in mesic forests between 400 and 800 m on Tahiti, while *S*. *St*.*-Johnianum* is a small endemic tree found on karstic and isolated Henderson Island [90]. The Marquesan species could not be determined from the wood charcoal [89]. The extension of this genus to the Marquesas is perhaps not surprising, as it fills a regional distribution gap.

### Arthropod assemblages

**Fig 11:**
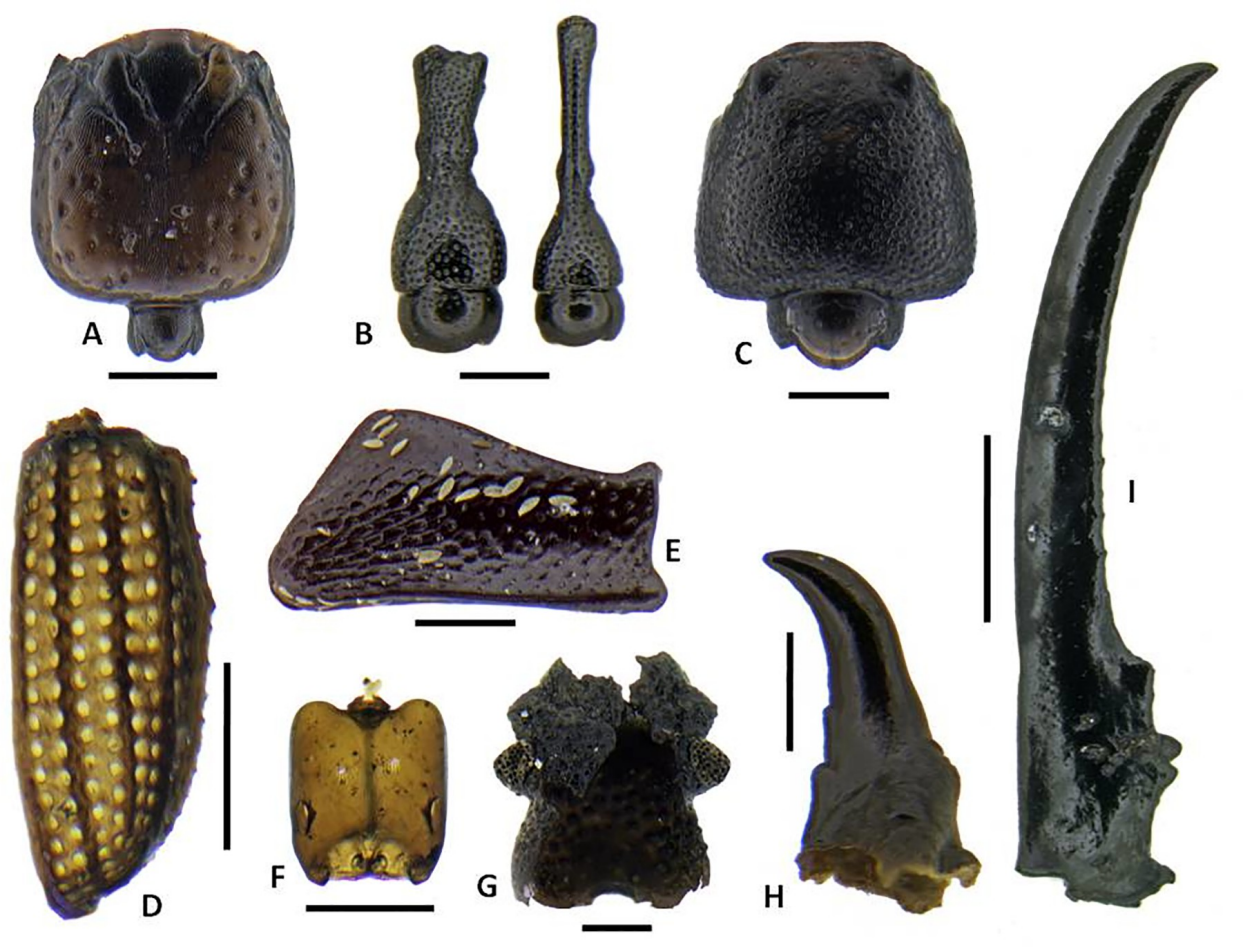
Representative insect subfossils of indigenous/endemic taxa 1. A. *Adamanthea* (Staphylinidae) beetle head; B. Cossonine ‘B’ weevil heads, male and female; C. Paederine staphylinid beetle head; D. *Mumfordia* (Latridiidae) beetle right elytron; E. *Ampagia* (Curcuclionidae) weevil hind femur; F. *Platyscapa* (Agaonidae) fig wasp head; G. *Proterhinus* (Belidae) weevil head; H. *Cryptotermes dolei* (Kalotermitidae) termite mandible; I. *Chelisoches morio* (Chelisochidae) earwig forcep. Scale bars: A-H, 0.25 mm. I, 2 mm. (Photo by N. Porch).

**Fig 12:**
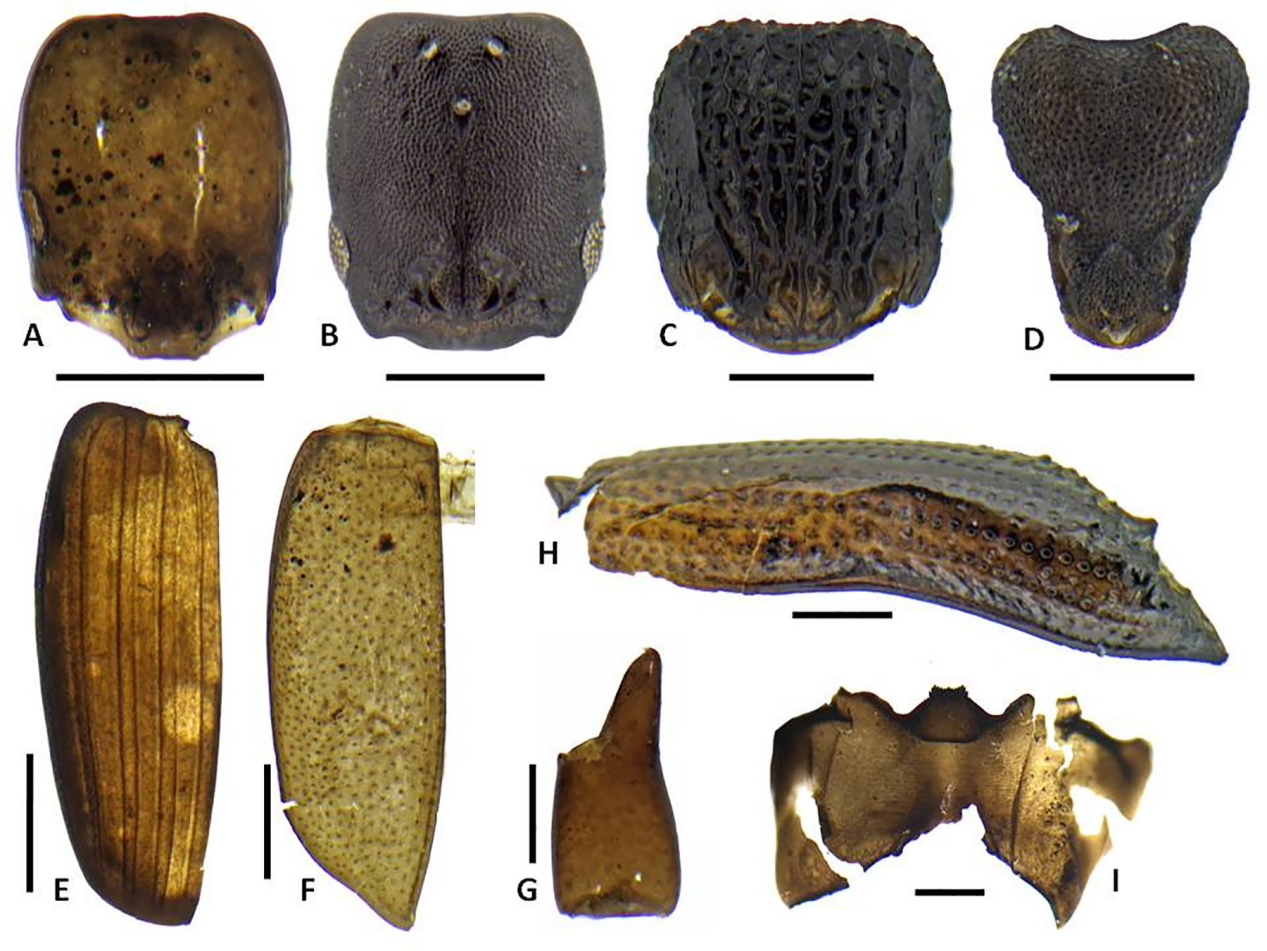
Representative insect subfossils of indigenous/endemic taxa 1. A. *Monomorium* sp. ant (Formicidae) head; B. *Ponera cf*. *incerta* queen ant (Formicidae) head; C. *Tetramorium* sp. 3 ‘new?’ ant (Formicidae) head; D. *Strumigenys cf*. *mumfordi* ant (Formicidae) head; E. Laemophloeidae beetle left elytron; F. *Liodessus*-type (Dytiscidae) beetle left elytron; G. Pseudoscorpion indet. chelicera; H. Xyleborini 1 (Curculionidae) weevil left elytron; I. *Simulium* (Diptera) larval head capsule. Scale bars all 0.25 mm. (Photo by N. Porch).

**Fig 13:**
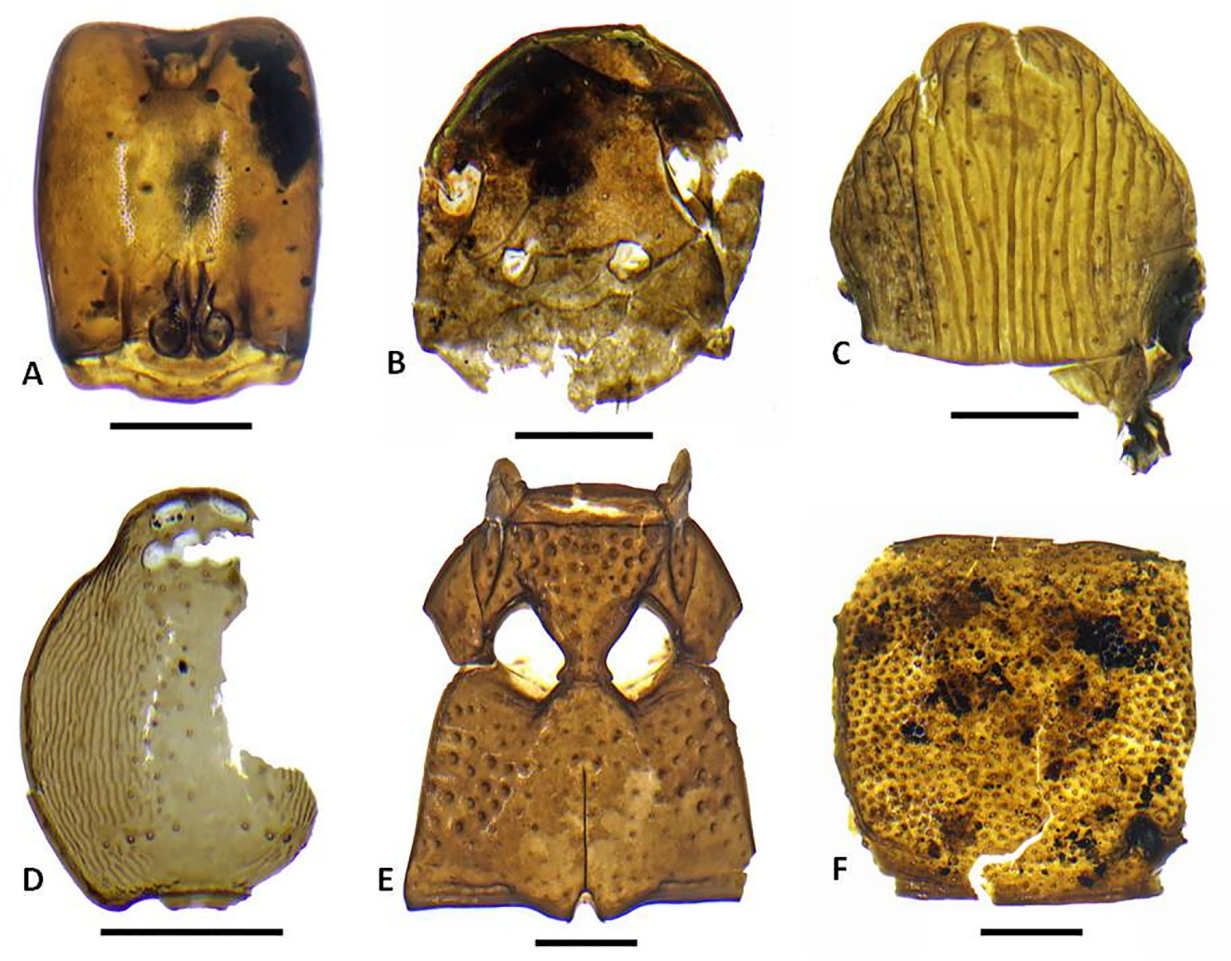
Representative insect subfossils of likely introduced taxa. A. *Hypoponera cf*. *punctatissima* ant (Formicidae) head; B. *Nylanderia sp*. ant (Formicidae) head; C. *Tetramorium bicarinatum* ant (Formicidae) queen pronotum; D. Opopaea-type spider (Oonopidae) spider carapace; E, F. *Cryptamorpha desjardinsii* (Silvanidae) (meso- and metaventrites (E) and prothorax (F). Scale bars all 0.25 mm. (Photo by N. Porch).

A total of 2124 identifiable arthropod remains were recovered from the five bulk sediment samples, dominated by 777 mites (not further sorted to morphospecies), 704 ants, 391 beetles and a smaller number of other insects and arachnids (Figs 11–13; Table 5). The most diverse group of insects are the beetles with at least 61 species representing at least 19 families, with a small number of taxa unassigned to a family. The ants include representatives of 11 species, two of which are singletons. The arachnids include at least one common pseudoscorpion represented by chelicerae, and several families of spiders represented by their distinctive carapaces. Other taxa that occur in small numbers include earwigs (Dermaptera), termites and cockroaches (Blattodea), wasps (Hymenoptera), larval, adult, and pupal flies (Diptera) and true bugs and scale insects (Hemiptera).

**Table 5:** Summary of identified arthropod material.

Group	Family/Group	Taxon	Acc. 7243	Acc. 7244	Acc. 7245	Acc. 7246	Acc. 7247	Total	Biogeography
**Mites**	‘Oribatida’	Oribatida	185	125	145	89	172	716	Various
	‘Mesostigmata’	Mesostigmata	16	29	8	2	6	61	Various
**Pseudoscorpions**	Indet.	Indet. (chelicera)	16	3	10	2	5	36	Various
**Araneae**	Sparassidae	Sparassidae A	1			1		2	Indig?
**Araneae**	Oonopidae	*Opopaea*-type	1			1	1	3	Indig./Intro?
**Araneae**		*Gamasamorpha*-type			1			1	Intro?
**Araneae**	Linyphiidae?	Linyphiidae				1		1	Indig?
**Araneae**	Indet.	Family A		1				1	Indig?
**Araneae**	Indet.	Family B	1	1				2	Indig?
**Coleoptera**	Carabidae	*Tachys sexguttatus* (Fairmaire)	1				1	2	Indig.
	Dytiscidae	*Liodessus*-type	1					1	Endemic
	Staphylinidae	*Adamanthea insularis* (Cameron)	5	2	1	1		9	Endemic
		Xantholinine 2				1		1	Endemic?
		*Philonthus*-type 1	1	2	1		1	5	Intro/Indig?
		Aleocharinae 1			1			1	Indig?
		Aleocharinae 2			1			1	Indig?
		Aleocharinae 3			1			1	Indig?
		Paederinae *Scopaeus*-type	3	7	4	1	1	16	Indig?
		Paederinae B	5	7	4	2	2	20	Indig?
		Paederinae C				1		1	Indig?
		Oxytelinae *Carpelimus*-type 1	8	4	4	1	6	23	Indig?
		Oxytelinae *Carpelimus*-type 2	9	1	3	2	3	18	Indig?
		Osoriinae *Clavilispinus* sp.	2	1	1		1	5	Indig?
	Ptiliidae	Ptiliid Genus A	1	3	1	1	5	11	Indig?
	Histeridae	Histerid Genus A				1		1	Indig?
	Hydrophilidae	*Omicrus* sp.larval head	1		1			2	Indig
		*Omicrus* cf. *brevipes* Sharp	1			1		2	Indig
	Elateridae	*Simodactylus* sp.1	1	1	1	1	2	6	Indig
		*Melanoxanthus* spp.		1	1	1		3	Endemic
	Ptinidae	*Dorcatomiella*	3	2	1	1	1	8	Indig.
		Ptinid Genus B	1	1				2	Indig?
	Latridiidae	*Mumfordia* sp.1		1		1	2	4	Endemic
	Nitidulidae	Nitidulid Genus A	1				2	3	Indig?
	Bostrichidae	*Tetrapriocera*? sp.	1					1	Indig?
	Silvanidae	*Cryptamorpha desjardinsii* (Guérin-Méneville)	2	1	1	1	2	7	Polynesian
		*Monanus* sp.1	3	1	1	1	1	7	Indig.
	Ciidae	*Cis* sp.1			1			1	Endemic
	Discolomatidae?	Discolomatidae			1		1	2	Unknown
	Laemophloeidae	Laemophloeid A	3	1	1	1	1	7	Endemic
		Laemophloeid B				1		1	Endemic
	Zopheridae	*Bitoma*-type		1		1		2	Endemic
		*Pycnomerus* spp.				1	1	2	Endemic
	Family indet.	Beetle A	2	1	2		1	6	Unknown
		Beetle B	1		1		1	3	Unknown
		Beetle C	1					1	Unknown
		Beetle D	1					1	Unknown
		Beetle E	1			1		2	Unknown
		Beetle F				1		1	Unknown
	Belidae	*Proterhinus* sp.				1		1	Indig./Endemic
	Anthribidae	Indet. fragments	6	2	3	1	1	13	Various
		Genus A		1				1	Indig./Endemic
		Genus B					1	1	Indig./Endemic
	Curculionidae	*Rhyncogonus*?	1	1	1	1	1	5	Island Endemic?
		Genus?				2		2	Unknown
		*Ampagia* ’Nuku Hiva’			1	1		2	Island Endemic
		*Miocalles*-type 1		1		1		2	Island Endemic
		*Miocalles*-type 2			1			1	Island Endemic
		Xyleborini 1	1	1	1	1	2	6	Indig./Endemic
		Xyleborini 2	2		1			3	Indig./Endemic
		Cossoninae A	1					1	Indig./Endemic
		Cossoninae B	30	30	19	20	21	120	Indig./Endemic
		Cossoninae C	1			1	1	3	Indig./Endemic
		Cossoninae D	1					1	Indig./Endemic
		Cossoninae E		2	1	1		4	Indig./Endemic
		Cossoninae F			1			1	Indig./Endemic
		Cossoninae G			1			1	Indig./Endemic
		Cossoninae H				1		1	Indig./Endemic
		Cossoninae J					1	1	Indig./Endemic
		Cossoninae K				1	1	2	Indig./Endemic
		Cossoninae L					1	1	Indig./Endemic
		*Hypothenemus* spp. (2)	5	10	3	4	6	28	Indig./Endemic
**Hemiptera**	Pentatomidae	Pentatomidae A	1					1	Indig./Endemic
	Anthocoridae?	Anthocorid-type	2				1	3	Indig./Endemic
	Reduviidae	Reduviidae (Emesinae) sp.				1		1	Endemic
	Coccidae	Coccidae A				1	1	2	Indig?
	Diaspididae	Diaspidid A							Indig?
	Aleyrodidae	Aleyrodid A							Indig?
**Hymenoptera**	Agaonidae	*Platyscapa* sp.	7	2	4	4	9	26	Indig.
	Bethylidae	*Sclerodermus* sp.	1	1	1			3	Endemic?
		Wasp heads indet	9	4	3	11	8	35	Various
	Formicidae	*Ponera* cf. *incerta* (Wheeler)	4		1	4	4	13	Indig.
		*Tapinoma* sp.1	52	65	65	18	21	221	Indig.
		*Strumigenys* cf. *mumfordi* (Wheeler)	2	3	2	1	2	10	Endemic
		*Monomorium* sp.	2		2		2	6	Indig.
		*Rogeria/Tetramorium* sp.					1	1	Polynesian?
		*Tetramorium* sp.3 ‘new?’			1	2		3	Endemic?
		*Tetramorium pacificum* Mayr	3	3	2	2	3	13	Indig.
		*Tetramorium bicarinatum* (Nylander)		2				2	Polynesian
		*Hypoponera* cf. *punctatissima* (Roger)	38	37	24	14	9	122	Polynesian
		*Nylanderia* sp.	89	49	62	60	52	312	Polynesian
		*Paratrechina longicornis* (Latreille)	1					1	Contamination?
**Diptera**		Adult fly heads indet.	2	1	1	1	4	9	Various
	Chironomidae	Chironomidae larva	5	2	6	4		17	Indig?
	Simuliidae	Simuliidae larva	1	1	3	3		8	Endemic
	Culicidae	Culicidae (*Culex* sp.)			4	6		10	Indig?
	Indet.	Diptera Pupa 1	3	1		1	1	6	Unknown
	Indet	Diptera Pupa 2	1		1			2	Unknown
	Indet	Diptera Pupa 3	1			1		2	Unknown
	Indet	Diptera Pupa 4		3	1			4	Unknown
	Indet	Diptera Pupa 5		2	3	3	2	10	Unknown
	Indet	Diptera Pupa 6					1	1	Unknown
	Ceratapogonidae	Ceratapogonidae pupa	2			3	23	28	Unknown
**Blattodea**	Kalotermitidae	*Cryptotermes dolei* (Light)	1	1	1		1	4	Endemic
	Indet.	Termite A	1	1				2	Unknown
		Termite B		1	1			2	Unknown
	Indet.	Cockroach A		1	1			2	Indig?
		Cockroach B				1	1	2	Indig?
**Dermaptera**	Chelisochidae	*Chelisoches morio* (Fabricius)	7	3	12			22	Indig.
	Anisolabididae	*Euborellia annulipes* (Lucas)	2					2	Polynesian
	Family?	Genus C	1					1	Indig.
	Family?	Genus D		1				1	Indig.
		**Totals**	**566**	**429**	**434**	**297**	**401**	**2124**	

The similarity of all five samples in terms of the major taxa present and their relative abundances implies there is little change in the local ecosystem during the period in which Layer IV was deposited. This is consistent with the radiocarbon results that, as noted above, suggest very rapid accumulation of this stratigraphic unit. Additionally, the arthropod remains are consistent with environmental conditions suggested by the associated plant materials.

## Discussion

It is well known that human colonists of East Polynesia transformed island landscapes in a myriad of ways, but the timing, particulars, and long-term ecodynamics are incompletely resolved. Transdisciplinary research and multi-proxy approaches like those undertaken here hold significant promise for improved understanding of complex socio-ecological relationships. The Ho‘oumi Beach findings inform on the timing of human arrival and offer a window onto early human activities and initiation of what became long-term impacts in one of region’s most remote archipelagos.

### Human arrival in the Marquesas Islands

Three independent proxies inform on the timing and nature of early human activity at Ho‘oumi Beach. Anthropophilic arthropods, taxa that are strongly associated with Polynesian activities elsewhere in the region, were found throughout Layer IV. Both contemporary biogeographic distributions, and occasionally their recovery from sediment cores with substantial prehuman records, have helped to establish the association of these taxa with Polynesian arrivals [19, 92]. Most likely these non-native arthropods were transported along with traditional Polynesian root crops, such as taro and yam. Microcharcoal particles are a second proxy of human activity, as natural fires are rare in the Marquesas Islands [59, p. 19). Microcharcoal occurs throughout Layer IV but is most abundant in the lower part of the deposit. Elsewhere on Nuku Hiva, dense charcoal deposits have been observed at the base of stratified cultural sequences, suggesting burning frequently accompanied initial use of coastal areas [93, 94]. In the Ho‘oumi case, the very small particle size suggests firing elsewhere in the valley, most likely upstream from Trench 1, and long-distance transport. A more direct indicator of human activity is the adzed timber of a locally extinct Sapotaceae species (Fig 10). This is probably an artefact of forest clearance but may have been purposefully shaped, as for example for use as a fence stake. In combination these three proxies provide a solid basis for inferring human activities coincident with the formation of Layer IV. The three proxies are also consistent with practices and outcomes relating to the establishment of gardens, including forest clearance (cutting and burning), erosion and sediment transport, and the establishment of Polynesian arthropods, some of which are commonly associated with cultivation.

Eleven ^14^C analyses allow for a robust age estimate of these anthropogenic markers and the other plant and arthropod subfossils. A sample from the base of the overlying, non-cultural, sedimentary unit (Layer III) provides a constraint for the Layer IV ^14^C results. Analyses by three laboratories identified two outliers and an inter-laboratory offset. The remaining eight analyses, with one exception, were run on three different kinds of short-lived materials to avoid inbuilt age [69, 70, 95]. A sample from the exterior of the small diameter timber (Wk-50151) returned a ^14^C result that is consistent with the short-lived material dates. These results place initial human activity in Ho‘oumi Valley between mid-11^th^ to early 13^th^ centuries AD. The Bayesian age model narrows the Layer IV age estimate to the mid-12^th^ century AD (*1129–1212 cal*. *AD*, 95.4% HPD).

These findings are usefully compared with other early ^14^C analyses, both at Ho‘oumi Beach and elsewhere on Nuku Hiva Island. Domestic activities in Anaho Valley to the north (Fig 1) are slightly later, dating between the mid-12^th^ to mid-13^th^ centuries AD at the Moetai site [96]. At Ho‘oumi Beach itself, nearby excavations on higher ground to the north identified a stratigraphically superior cultural occupation that Bayesian analysis places between the 13^th^ and 14^th^ centuries AD [63]. Thirteenth to 14^th^ century occupations are also found in other northern valleys (Hakaea and Hatiheu), and continue at Anaho [93, 94]. Although Ha‘atuatua Valley (see Fig 1) on the eastern coast of Nuku Hiva has long been considered an early Marquesan settlement area, only one ^14^C result from the lowest occupation (Layer D) comes close to meeting current ^14^C standards and this was on unidentified wood charcoal (970 ± 70) [97; reviewed in 96, p.7]. The age estimate of that sample is slightly earlier than the results reported here.

Elsewhere in the archipelago, 12^th^ to 13^th^ century dates come from occupation sites on the islands of Ua Huka (Hane and Hokatu sites) [98–100], Eiao [101, p. 78], and Tahuata (Hanamiai site) [33]. Many of these ^14^C results are on unidentified wood charcoal raising some uncertainty about the accuracy of the age estimates [96]. With eleven ^14^C analyses, Hane is the best dated and most thoroughly studied of these [98, 99, 101–104]. Here, however, the individual conventional radiocarbon dates (CRAs) from the basal cultural layer range from 810 ± 40 to 1088 ± 25 [99, Table 1]. Results on a twig, a definitively short-lived material (Beta-260938, CRA 810 ± 40), calibrates to AD 1226–1280 (68.2%, SHCal20), which is slightly later than the Bayesian model estimate reported here for Ho‘oumi (*1129–1212 cal*. *AD*, 95.4% HPD). A second Hane sample on unidentified broadleaf charcoal (Wk-27331, 928 ± 30) overlaps with the Ho‘oumi results.

Overall, the Ho‘oumi chronometric results are robust with respect to the materials dated (short-lived), the number of samples analysed, and the implementation of interlaboratory comparisons. The internally consistent series modestly extends the Nuku Hiva cultural sequence and is not at odds with results from elsewhere in the archipelago.

The Ho‘oumi evidence is regionally significant as well, demonstrating that human colonisation of the Marquesas Islands was pene-contemporaneous with Polynesian arrival in the Society Islands to the east. The Fa‘ahia site on Huahine Island, is dated by eight ^14^C results on short-lived materials [105, Table 2]. Recalibration of those CRAs using the newly available SHCal20 curve [73] places initial occupation at AD 1044–1265 (maximal age range of the 68% probabilities). There is some uncertainty around the early end of the Fa‘ahia distribution; relative areas under the 68% probability distributions only reach 0.513 or less, and in half the cases a mid- to late 12^th^ century age is indicated as more likely. This uncertainty leaves open the possibility that human settlement in the Marquesas Islands was contemporaneous with, or soon after, that in the Societies.

Results from both the Societies and the Marquesas, however, post-date initial human activities in the southern Cook Islands, where Sear and colleagues [106] proposed that exploratory expeditions preceded full-scale settlement. Lake core sediments on Atiu Island produced mammalian faecal sterols dating to ca. the 10^th^ century AD, followed by more significant environmental disturbances roughly a century later. Consistent with the Atiu Island model, Kirch and colleagues date the basal cultural occupation of the Tangatatau Rockshelter, on nearby Mangaia Island, from the 11^th^ century AD [107].

The Ho‘oumi results, along with the forgoing archaeological chronologies from the northern Marquesas Islands, point to a longer occupation sequence relative to that recently proposed by Ioannidis and colleagues [108]. Drawing on genome-wide data and novel computational analyses, they suggest divergence (and by extension settlement) dates for the northern Marquesas of around AD 1330–1360. Notably, these divergent dates are suggested to be *terminus ante quem* for a given settlement locality. As suggested by the foregoing review, chronometric assessments for the northern Marquesas, and Nuku Hiva in particular, are more in line with their proposed divergence estimates for the southern Marquesas (Fatu Hiva), Tuamotu, and Austral Islands. Along with indicating a deeper human history for the northern Marquesas, our results raise questions about the processes that have affected the genetic model outcomes [108] relating to the northern group, such as the potential of post-settlement trade contact [63, 96, 108 p. 524–25].

### Stratigraphic integrity of the Ho‘oumi deposit

The ^14^C evidence also informs on the stratigraphic integrity of the Ho‘oumi deposit. AMS results from throughout the unit failed to identify any stratigraphic inversions. For the arthropods, the recovered remains were typically compressed, consistent with having been preserved under pressure within a sedimentary sequence. Additionally, there is very limited (if any) evidence of contamination suggested by the biotic materials themselves. In the case of the seeds, fruits, wood, and microfossils, no definitive post-western contact taxa were identified. Although there is the possibility that one or more of the specimens identified to genus could include historic introductions, this is considered unlikely (see below). Among the arthropods, the only potential example of a modern contaminant is a single specimen of *Paratrechina longicornis*—a species that is most likely a post-contact introduction to Polynesia [61]. These factors suggest extremely low levels of disturbance or contamination by more recent materials.

### Botanical assemblages

This is the first consequential record of the pre-European herbaceous (non-woody) vegetation for the Marquesan Islands. A previously analysed sedimentary core from the Nuku Hiva interior (To‘ovi‘i Plateau at 810 m) identified a small number of arborescent taxa, herbs, and ferns dating from ca. 1350–1400 AD—but the core was generally pollen-poor [109]. In contrast, a diverse range of taxa have been identified from the Ho‘oumi site, with two communities potentially represented: low to mid-elevation moist-wet forest (following Florence and Lorence [44]) and coastal forest. Elements of the former include both canopy and understory taxa. Among the potential canopy dominants are *Pandanus tectorius*, *Pterophylla* (syn.*Weinmannia*) cf. *marquesana*, *Cyrtandra* cf. *thibaultii*, and *Angiopteris marchionica*, with an herbaceous fern understory also suggested. Florence and Lorence [44] place this forest community today between 300 to 800 m elevation. While the Ho‘oumi examples conceivably could have been fluvially transported from the valley interior, the possibility that some of these taxa extended to lower elevations is equally plausible. Similarly, the *Metrosideros* pollen intimates that this taxon, now restricted to higher elevations [44, p. 234], once grew at lower altitudes. Supportive evidence comes from Anaho Valley to the north where *Metrosideros* wood was recovered from an early fire feature [23].

Comparisons with archaeobotanical assemblages from nearby but chronologically later strata that are associated with residential features and domestic activities [63] are instructive and give insights into the vegetation transformations that accompanied Polynesian settlement over time. In these later contexts, *Calophyllum*, *Thespesia*, *Cordia subcordata*, *Gymnosporia crenata*, *Phyllanthus marchionicus*, *Alstonia costata*, and *Sapindus saponaria* were identified. Several Polynesian introductions are also represented, such as *Aleurites moluccanus* (candlenut), *Casuarina equisetifolia* (ironwood), cf. *Artocarpus altilis* (breadfruit), and cf. *Spondias dulcis* (Otaheite apple), taxa that are suggestive of house gardens or nearby cultivations.

### Arthropod records

The arthropod assemblage is the first pre-western record of its kind for the Marquesas Islands. It too was diverse, with more than 100 distinct taxa representing at least nine major groups and more than 39 families. Biogeographic assignments are in some cases tentative given the possibility of extinct taxa, the limited knowledge of some native Marquesan biotic groups, and the possibility of previously unrecognised Polynesian introductions. Taxa of aquatic environments include relatively infrequent simuliid, chironomid, and ceratopogonid flies. Among the more unusual finds is a single elytron of a dytiscid water beetle that belongs to a genus that has not previously been recorded for eastern Polynesia (cf. *Liodessus*). The tribe (Bidessini) is known only from living material collected on Raiatea and from abundant fossils of *Allodessus* and another unidentified genus (not *Liodessus*) from other island subfossil records [19, 110].

The simuliids indicate flowing water but may derive from upstream rather than the immediate locality if the local environment of the site today was similar in the past. The relative infrequency of taxa indicating aquatic environments suggests the presence of a marsh at the time of deposition, rather than open water. This is supported by the abundance and diversity of terrestrial taxa, which comprise the bulk of the assemblage in terms of both numbers of individuals and diversity of taxa. The absence of acidocerine hydrophilids (*Enochrus/Chasmogenus*) is notable given their ubiquity in other eastern Polynesian subfossil assemblages, where they can be abundant in terrestrial-dominated insect assemblages that accumulated in marsh settings. However, Ramage [55] does not list *Enochrus* as occurring in the Marquesas, nor *Chasmogenus* as occurring is French Polynesia. These contrasts probably reflect a combination of large scale changes that have occurred in the nature of aquatic habitats, and therefore assemblages, over the past 800–1000 years, and the relative lack of extensive targeted surveys for many groups of non-marine invertebrates, including aquatic beetles.

The terrestrial arthropod species are dominated by indigenous taxa of forest habitats, with a large variety of saproxylic species that are associated with dead and decaying plant matter. Many of these groups are widespread across eastern Polynesia. Of the 391 beetles, the majority belong to saproxylic taxa. These include an abundant and diverse range of cossonine weevils that are commonly encountered in fern and palm stems or the sapwood of dead trees. Because the modern cossonine fauna of eastern Polynesia is poorly known, it is difficult to be more specific about both the taxonomic composition of the fauna and the detailed ecology of the species. Other weevils are also present, including *Ampagia*, *Miocalles*, and the belid *Proterhinus*, and likely *Rhyncogonus*, which is represented only by distinctive large tarsal segments. Species of these taxa are frequently collected from living plants but may not necessarily be obligate to specific plant taxa. All of the above genera have been previously recorded from the Marquesas, with a small number of endemic species in the first three and many endemic species in *Rhyncogonus* [55].

Bark beetles, which generally prefer living trees, are represented by two species of Xyleborini and by at least two species of the genus *Hypothenemus*. Further saproxylic beetles include the bostrichid, elaterids, zopherids, laemophloeids, ptinids and other taxa listed as saproxylic in Table 5. The presence of at least two species of laemophloeid is notable given that this family is not currently known to contain indigenous/endemic taxa in the Marquesas [55]. It is unlikely these taxa are Polynesian introductions as they fit well with the overall domination of the insect assemblage by saproxylic taxa.

In addition to these saproxylic taxa are a range of less common but diverse predators, detrivores, fungivores, and hygrophiles. The spore-feeding latridiid genus *Mumfordia* is known from single endemic species found on Hivaoa and Ua Huka [111]. The record here is likely a first for Nuku Hiva and almost certainly a new species. Species that occur around standing waterbodies include the carabid *Tachys sexguttatus* and the staphylinid *Carpelimus*, which is represented by several species but very poorly known in the region. The detrivorous hydrophild *Omicrus* has not been recorded from the Marquesas but is almost certainly overlooked in the modern fauna because of its very small size. The consistent presence of *Platyscapa* fig wasps, pollinators of *Ficus*, corresponds well with the abundance of fig seeds in the plant assemblage.

As a whole the arthropod assemblage is consistent with the presence of a relatively intact indigenous lowland forest. Also of note, the Ho‘oumi assemblage includes beetle taxa like *Adamanthea* (Staphylinidae), *Ampagia* (Curculionidae), *Bitoma*, and *Pycnomerus* (Zopheridae) that, to our knowledge, have not previously been collected on Nuku Hiva and therefore may represent new records (i.e., *Adamanthea*) and new species (i.e., *Ampagia*, *Mumfordia*, and the zopherids). Detailed survey is needed to determine whether these, and indeed many of the other saproxylic taxa, persist on Nuku Hiva, most likely at higher elevations. It should be noted that many such taxa, including *Pycnomerus* and other zopherids, and *Ampagia*, are present in prehuman insect assemblages of the eastern Pacific islands but rapidly disappear soon after the arrival of Polynesians and the attendant transformations of lowland forests [19, 32]. In light of these regional findings, the Ho‘oumi record most likely signals some of the earliest early human activities in this valley.

### Polynesian translocations

Lacking more comprehensive reference collections, definitive taxonomic determinations of the seed and fruit macrofossils have been challenging and it is uncertain if Polynesian translocations are represented. Nonetheless, a small number of specimens warrant discussion. The two cucurbit seeds (Cucurbitaceae) are intriguing. Morphologically they are broadly inconsistent with the South American-derived *Lagenaria siceraria* (bottle gourd). Whistler [20], however, notes another gourd, *Benincasa hispida* (wax gourd, *hoe puo*), that was collected in the Marquesas as early as 1838 and Brown [35] considered the species a possible Polynesian introduction. Informal experiments by Lewis on modern marginate seeds of *B*. *hispida* show that spongy surface tissue often degrades to a state consistent with the irregular surface of the Marquesan subfossils. *Zehneria mucronata* is another potential candidate; this taxon is considered indigenous to the Society Islands and the Marquesas, today being found in montane cloud forest [47]. Modern seeds of *Zehneria mucronata* were not available for comparison but based on published materials the seeds of this species appear smaller than the subfossils. Unfortunately, the two Ho‘oumi specimens are poorly preserved and neither could not be convincingly attributed to a specific genera.

The *Solanum* specimens are also of interest. The subfossil seeds do not match *S*. *americanum* or *S*. *opacum*, which are in the same taxonomic complex. Rather, the morphology of the Ho‘oumi seeds is closer to that of *S*. *repandum*. The origin of this *Solanum* is uncertain but it is often found in association with human activities and is distributed to several central Polynesian archipelagos, including Fiji, Sāmoa, Niue, the Cook Islands, and Tahiti [20, pp. 192–93]. In the Marquesas, Wagner and Lorence [81] consider *S*. *repandum* to be indigenous, though they note it is found around villages, from 0–100 m elevation. The fruits were eaten across Polynesia and in Tahiti the leaves are used to make a red dye; medicinal use has also been recorded [20, pp. 192–93].

*Morinda citrifolia* is often considered a Polynesian dispersal. In East Polynesia, the medicinal properties of the fruits are valued and the roots and bark produce colourful dyes [20, p. 149]. In a recent wood charcoal study involving a large number of samples from three northern Nuku Hiva valleys, *Morinda* was rare and restricted to late contexts. On these grounds, Huebert and Allen [36] proposed it may have been a secondary introduction to the Marquesas, post-dating colonisation by several hundred years. The macrofossil remains from Ho‘oumi now extend the temporal depth of this taxon into the early settlement period, a finding that fits with previous observations of *Morinda citrifolia* on Rimatara (Austral Islands) in contexts that pre-date human arrival. Our results raise the possibility that it also reached the Marquesas Islands in pre-human times.

The Ho‘oumi arthropod record is likewise dominated by indigenous taxa. However, a small number of species were most likely introduced, inadvertently, by Polynesians. These include the beetle *Cryptamorpha desjardinsii*, the earwig *Euborellia annulipes*, and at least three ants, *Hypoponera* cf. *punctatissima*, *Tetramorium bicarinatum*, and *Nylanderia* sp. Both *C*. *desjardinsii* and *E*. *annulipes* can be common in Polynesian-aged sediments elsewhere in Polynesia [19]. The ant genera *Hypoponera* and *Nylanderia* are among the most abundant insect taxa in the Ho‘oumi deposit, a pattern that frequently characterises invasive taxa. These taxa are common in Polynesian-aged sediments elsewhere in the region, and have not been recovered from pre-human contexts [17, 19]. Both genera are considered introduced to French Polynesia by Ramage [61]. Two other ant taxa that are introductions are infrequent. *Tetramorium bicarinatum* occurs elsewhere in eastern Polynesia in Polynesian-aged sediments, suggesting it is potentially also a Polynesian introduction to the Marquesas, although it is considered a historical introduction by Ramage [61]. The single specimen of *Paratrechina longicornis* may be a contaminant introduced during sampling as it is not known from prehistoric sediments anywhere in Polynesia and Ramage [61] considers it a post-contact (historic) introduction.

### Extinctions and extirpations

Both the plant and arthropod assemblages suggest major transformations of Marquesan lowland environments over time, with changes in both species compositions and relative abundances. The clearest example is provided by *Sideroxylon*, only recently recognised as a now extinct and apparently once widespread and abundant component of lowland Marquesan forests [23]. The distribution of *Sideroxylon* now extends to Ho‘oumi Valley, but it is restricted to Layer IV, where other extinctions are suggested by the material reported here.

Other possible extinct taxa (or extirpated, in the case of *Trema*) include *Mussaenda*, *Macaranga*, *Trema*, and *Acalypha*, none of which have been previously recorded for Nuku Hiva or for the archipelago, with the exception of *Trema*, which has recently been identified on Ua Pou. Two *Mussaenda* species are currently known for Marquesas but both are cultivars and neither produce seed [81, 112, 113]. Indigenous *Mussaenda* are found elsewhere in Melanesia, Western and Eastern Polynesia, as for example *M*. *raiateensis*, which occurs on Raiatea (Society Islands) where it is extremely rare, and limited to montane cloud forest [114, also J.-Y. Meyer, pers. com., 2021], and thus a Marquesan representative is not unexpected.

Characters of the subfossil *Mussaenda* seeds are intermediate between those of the two extant Pacific species, *M*. *cylindrocarpa* and *M*. *raiateensis*, both in seed length and in number of pits per cell (Table 6). However, modern seeds have not been examined to assess whether the subfossils fall at the extreme end of the range of the variability for either species. Both modern species occur in forest edges from sea level to 1000 metres [115]. *M*. *cylindrocarpa* occurs mostly in Papua New Guinea and across the Pacific to Vanuatu on Erromanga Island [115]. *M*. *raiateensis* occurs in Vanuatu, Fiji, Tonga, Sāmoa, Wallis and Futuna, Cook and the Society Islands, the latter being the current eastern-most range. Thus the new Ho‘oumi subfossil record extends the eastern terminus of the genus *Mussaenda* in the Pacific.

**Table 6:** Morphometric data for two modern *Mussaenda* species (Rubiaceae) from Gideon [115] and for measurable Ho‘oumi subfossils.

Taxon	Seed size	Aperture no. per alveolae
***Mussaenda raiateensis* J.W.Moore**	0.9–1.5 mm long	8–15
***Mussaenda cylindrocarpa* Burck**	0.4–0.5 mm long	1–6
**Subfossils: sample 7247 (n = 2)**	0.7 mm long, 0.52 mm wide;	4–9
	0.65 mm long, 0.4 mm wide	
**Subfossils: sample 7244 (n = 1)**	0.6 mm long, 0.4 mm wide	4–7

Similarly, although there are no known indigenous Marquesan *Macaranga*, there are multiple species elsewhere in the region, some endemic. The Austral Island sedimentary cores again suggest one or more native species that were widespread prior to human arrival, highlighting the genus’ vulnerability to anthropogenic activities [19]. The extant French Polynesian *Macaranga* species are found in coastal, ridge, mesic, wet or montane cloud forests [79]. They are island or archipelago endemics [79], as island radiations are common within the genus [116]. The Ho‘oumi record extends the taxon’s range in the Pacific, from the Society Islands to the Marquesan archipelago.

*Acalypha* has also not been previously recorded in the Marquesas. Of the three French Polynesian taxa, two have larger seeds than the subfossils, and one has very similar dimensions to the subfossil, *Acalypha rapensis*, a Rapan endemic [79]. Notably the Marquesas Islands have floristic affinities with Rapa, sharing some 27 species [44]. The Ho‘oumi subfossil thus might represent the extirpation of *A*. *rapensis*, or alternatively could be an extinct Marquesan endemic.

The Marquesan endemic *Claoxylon ooumuense* was considered for the determination of the likely *Claoxlyon* subfossil. While of similar dimensions to the Ho‘oumi example, and with irregularly rugose-tuberculate sculpture on the dorsal face, *C*. *ooumuense* is excluded on the basis that its seed is smooth on the ventral surface and its shape is obovoid [figure 7.8 in 79], unlike the subfossil (Fig 9). The subfossil compares well to the line drawing and description of *Claoxylon collenettei*, an endemic of Rapa [79, pp. 56–8, figure 6.8] and again may represent an extirpation of *C*. *collenettei* or an extinct Marquesan taxon.

*Trema* is yet another possibly indigenous and now extirpated Nuku Hivan taxon; its seeds were found throughout the Ho‘oumi deposit. Florence [79] considered *T*. *discolor* a French Polynesian endemic and it was well represented in a recent lake core study on Mo‘orea (Society Islands) [117]. In the Marquesas, in contrast, there is only a single recorded occurrence of *Trema*, collected on Ua Pou in 2003, initially leading Wagner and Lorence [81] to propose it was a naturalised example of *T*. *orientalis*, but they now consider it indigenous [47].

The *Pritchardia* (palm) seed fragments are of particular interest. In their review of East Polynesian *Pritchardia*, Butaud and Hodel [91] note early historic accounts of the genus on Nuku Hiva, and specifically in Ho‘oumi but no wild specimens have been recently observed on the island. Two species were introduced to the Marquesas in historic times: *P*. *pacifica* probably arrived in the late 19th or early 20th century and *P*. *thurstonii* in the second half of the 20^th^ century [91, p. 151]. Our archaeologically recovered materials are the first pre-18^th^ century examples of the genus on Nuku Hiva; they confirm this taxon was native to the island, with the recently described *P*. *tahuatana* from Tahuata Island [91] being a possible species. However, an extinct *Pritchardia* could also be represented. The Ho‘oumi subfossils, fragments of seeds, are slightly larger than those of *P*. *pacifica*. The leaves and other parts of this palm were highly valued by Marquesans—for fine weaving, thatch on elite houses, and use in rituals, but the fruits may also have been favoured by the introduced Polynesian rat (*Rattus exulans*). Today Marquesan *Pritchardia* are extinct in the wild but Butaud and Hodel [91] suggest the genus was once an element of mesic to wet lowland forests.

It is difficult to assess the likelihood of insect extinctions here given that there has been little modern collecting specifically focussed on groups that might be missing from the modern biota. Elsewhere in eastern Polynesia the composition of the subfossil record has driven the collecting focus (e.g., for the genus *Pycnomerus* in the Austral islands) [32, 118], providing a better idea of what is likely extinct and what may be extant. Notable occurrences in the Ho‘oumi record, specifically groups not recorded in Ramage [55] or other literature, include the beetle genera *Mumfordia*, *Ampagia*, *Miocalles*, *Monanus*, the two unidentified laemophloeid taxa, and likely many of the unidentified cossonine weevils. The previously unrecorded species of water-beetle (*Liodessus*-type) is certainly a new genus record for the archipelago and for French Polynesia, although the single elytron does not allow much to be said of possible relationships. The presence of an ant species (*Tetramorium* sp. 3 ‘new?’) that cannot currently be identified demonstrates that the loss of taxa may extend to groups other than beetles. The history of discovery of one group of trogossitid beetles in French Polynesia, first in the lowland subfossil record of the Austral and Society Islands [17, 119], and their subsequent recognition in several collections from high elevation sites on Tahiti, suggests caution needs to be the rule when relatively good quality, high-elevation habitats remain, as on Nuku Hiva. Although it should be noted that even in this latter circumstance it is still likely that the majority of species that were endemic to low elevation islands, or restricted to low elevations of high islands, are now extinct.

These losses and transformations are still poorly understood but contemporary studies highlight the dynamic and recursive feedback relationships between vegetation communities, predators, pollinators, and nutrient recyclers. Some plant species, for example, played more pivotal roles, with Adamson [59, pp. 40–41] observing that *Metrosideros collina* and *Pterophylla marquesana*, which were both common Marquesan tree species, supported particularly large insect faunas relative to other arborescent taxa. In this regard he also drew attention to *Cyrtandra*. Exploration of these relationships over time will require more detailed identifications and additional assemblages, but those at hand clearly demonstrate that Marquesan biodiversity once exceeded that which is currently recognised.

## Conclusions

The anaerobic deposit at Ho‘oumi Beach (Nuku Hiva Island) sheds new light on human activities and the indigenous flora and fauna of the Marquesan lowlands at, or shortly after, initial human arrival. The adzed timber, micro-charcoal, and rapidly deposited, biotic-rich sediments are consistent with, respectively, forest clearance, burning, and erosion—the kinds of landscape modifications that commonly accompany the initiation of agriculture practices in pristine landscapes. Polynesian-associated arthropods, including a beetle (*Cryptamorpha desjardinsii*,), an earwig (*Euborellia annulipes*), and at least three genera of ants (*Hypoponera* cf. *punctatissima*, *Tetramorium bicarinatum*, and *Nylanderia* sp.) provide further evidence of the human fingerprint. Two plant taxa are conservatively considered native, *Morinda* and Cucurbitaceae, but the possibility that they are Polynesian introductions cannot, on present evidence, be discounted.

The well-preserved plant materials from Ho‘oumi facilitated high resolution ^14^C dating—a necessity when unravelling the relatively short human histories of East Polynesia. The dating of fruits and seeds avoided the problem of inbuilt age, which is often associated with wood charcoal from mature and/or long-lived trees, as might be expected on pristine landscapes. The resulting AMS and Bayesian analyses, in combination with the evidence for Polynesian-introduced arthropods, place human colonists on Nuku Hiva, and in the archipelago generally, in the mid-12^th^ century (*1129–1212 cal*. *AD*, 95.4% HPD). These new ^14^C results indicate human colonisation of Nuku Hiva was roughly two centuries earlier than recent genetic estimates [108], within a few decades of the Society Islands [105] if not contemporaneous, but after initial Polynesian explorations in the southern Cook Islands [106, 107].

Although the floral and faunal records remain to be fully identified, and may include a number of undescribed species, they make important contributions to our understanding of pre-human biodiversity in this archipelago, and the ecosystem changes that followed human arrival. The plant macro- and microfossil records suggest potential extinctions and point to taxa currently constrained to higher elevations that may have once extended into the Marquesan lowlands. The inventory of pre-western arthropods is an important first, with several new records for the archipelago and one for the cultural historical region of central East Polynesia at large. In time, these records stand to inform more fully on regional scale taxonomic relations and processes of adaptive radiation.

The Marquesan arthropod records also contribute to a growing body of evidence from across East Polynesia [17, 19, 32] relating to the diversity of invertebrates that accompanied human settlement. Some of the taxa identified at Ho‘oumi were potentially aggressive invasive species (e.g., Polynesian ant introductions), that probably spread with speed and had far-reaching ecological effects. These taxa, and others, likely had direct and indirect impacts on the native arthropods, food resources of native avifauna, lowland forest decomposition processes, and biogeochemical cycling.

The Ho‘oumi record underscores that biodiversity loss arose not only from human hunting, as often assumed based on terrestrial vertebrate assemblages, but also from habitat loss and the introduction of non-native species, large and small. The mid-12^th^ century anthropogenic activities observed at this coastal locality (forest clearance, firing, and dispersal of anthropophilic species) portend the more consequential human footprints that followed, in this valley, on Nuku Hiva, and probably elsewhere in the archipelago. As the anthropogenic niche expanded, Marquesan biodiversity was reduced, ecological relations realigned, and biotic communities re-structured. Although these long-term process have yet to be fully revealed, the rich biotic records presented here deepen understanding of the exceptional biodiversity of the remote Marquesan Islands and provide an ecological baseline for contemporary conservation efforts.

## Supporting information

S1 Text OxCal (ver.4.4.4) Bayesian model code. (DOCX)

S1 File Inclusivity in global research questionnaire. (DOCX)
