# Supplementary material for: Lost bioscapes: Floristic and arthropod diversity coincident with 12th century Polynesian settlement, Nuku Hiva, Marquesas Islands
Source: PLoS One. 2022 Mar 30;17(3):e0265224. doi: 10.1371/journal.pone.0265224 (PMC8967401; doi:10.1371/journal.pone.0265224)
Supplement: S1 Text — (DOCX) [file pone.0265224.s001.docx]

**Supporting Information**

**Lost bioscapes: Floristic and arthropod diversity coincident with 12^th^ century Polynesian settlement, Nuku Hiva, Marquesas Islands**

Melinda S. Allen, Tara Lewis, Nick Porch

**S1 Text. OxCal (ver. 4.4.4) Bayesian Model Code**

Plot()
 {
  Outlier_Model("Charcoal",Exp(1,-10,0),U(0,3),"t");
  Curve("SHCal20","shcal20.14c");
  Sequence()
  {
   Boundary("Start IV");
   Phase("IV")
   {
    R_Combine("pandanus 1")
    {
     R_Date("Wk-50153", 936, 25);
     R_Date("UCIAMS-230702", 955, 20);
    };
    R_Date("Wk-49524", 909, 24);
    R_Combine("cocos_1")
    {
     R_Date("Wk-50154", 930, 25);
     R_Date("UCIAMS-230703", 975, 20);
    };
    R_Date("Wk-50151", 933, 25)
    {
     Outlier("Charcoal", 1);
    };
    R_Date("Wk-50152", 905, 25);
    R_Date("Wk-49523", 928, 26);
   };
   Boundary("End IV/Start III");
   Phase("III")
   {
    R_Date("Wk-50155", 886, 25);
   };
   Boundary("End III");
  };
 };

**References**

Bronk Ramsey C. OxCal ver. 4.4.4 Online calibration program; 2021.

Hogg A, Heaton TJ, Hua Q, Palmer J, Turney C, Southon J, et al. SHCal20 Southern Hemisphere calibration, 0-55,000 years cal BP. Radiocarbon. 2020; 62(4), 759–778. <https://doi.org/10.1017/RDC.2020.59>
