# Supplementary material for: Lost bioscapes: Floristic and arthropod diversity coincident with 12th century Polynesian settlement, Nuku Hiva, Marquesas Islands
Source: PLoS One. 2022 Mar 30;17(3):e0265224. doi: 10.1371/journal.pone.0265224 (PMC8967401; doi:10.1371/journal.pone.0265224)
Supplement: S1 File — (DOCX) [file pone.0265224.s002.docx]

Inclusivity in global research

PLOS’ policy on inclusivity in global research aims to improve transparency in the reporting of research performed outside of researchers’ own country or community and ensures that PLOS publications reporting global research adhere to high standards for research ethics and authorship. Authors of relevant research articles may be asked to complete the questionnaire below, which outlines ethical, cultural, and scientific considerations specific to inclusivity in global research. This questionnaire may be requested when researchers have travelled to a different country to conduct research, if research uses samples collected in another country, research with Indigenous populations or their lands, or if research is on cultural artefacts. Researchers travelling to another country solely to use laboratory equipment will not normally be required to complete the questionnaire. However, the questionnaire can be requested at the journal’s discretion for any submission – if you have been requested to complete this questionnaire by the PLOS journal you submitted to, please do so.

Please complete the questionnaire below and include this as a Supporting Information file with your manuscript. Note that if your paper is accepted for publication, this checklist will be published with your article in the supporting information files. Please ensure that you reference the checklist in the main body of your manuscript. We suggest adding a subsection ‘Inclusivity in global research’ to your Methods section and adding the following sentence: “The questions have been designed to be applicable to a wide range of study types, and there are subsections for both human subjects research and non-human subjects research. If any of the questions are not relevant to your research please mark them as “N/A” as appropriate.

**Ethical considerations, permits and authorship**

*This section is applicable to all research types.*

Provide details as to who granted permissions and/or consent for the study to take place in the Methods section of your manuscript. This should include the names of **all** ethics boards, governmental organizations, community leaders or other bodies that provided approval for the study. If individuals provided approval refer to these people by their role or title but do not list their name(s).

Reported on page number 9 and in the Acknowledgements. Permissions were granted by the Gouvernement de la Polynésie Française, Ministère de la Culture et de l'Artisanat, with authorisation from the Délégation à la recherche (de la Polynésie française), and aided by the Service de la Culture et du Patrimoine (permit required), the Mayors of Taiohae and Hatiheu (approval required before approval of permit), and the French Polynesian Government Representative on Nuku Hiva, who gave permission to carry out excavations on government land.

If there were any deviations from the study protocol after approval was obtained please provide details of these changes in the Methods section of your manuscript.
Did this study involve local collaborators that are residents of the country where the research was conducted or members of the community studied? If you do not have any authors from said communities, please provide an explanation for this below.

no deviations

Everyone listed as an author should meet PLOS’ criteria for authorship and all individuals who meet these criteria should be included in the author byline, rather than the acknowledgements. Authorship criteria is based on the International Committee of Medical Journal Editors (ICMJE) Uniform Requirements for Manuscripts Submitted to Biomedical Journals - for further information please see here: <https://journals.plos.org/plosone/s/authorship>.

We had local collaborators who assisted with field logistics and paid local field assistants who were involved in the field study. However, these individuals were not involved in scientific analyses reported here, and did not meet the PLOS criteria for authorship; they are thanked in the Acknowledgements for their respective contributions.

**Human subjects research (e.g. health research, medical research, cross-cultural psychology)**

Did you obtain written informed consent from a representative of the local community or region before the research took place? How did you establish who speaks for the community? Details of written informed consent obtained from study participants should be reported separately in the Methods section of your manuscript.

This study did not involve human subjects research.

How did members of the local community provide input on the aims of the research investigation, its methodology, and its anticipated outcome(s)?

Not applicable as this study did not involve human subjects research.

When engaging with the local community, how did you ensure that the informed consent documents and other materials could be understood by local stakeholders?

Not applicable as this study did not involve human subjects research.

Will the findings of the research be made available in an understandable format to stakeholders in the community where the study was conducted (e.g. via a presentation, summary report, copies of publications, etc.)? Please provide details of how this will be achieved.

Not applicable as this study did not involve human subjects research.

**Non-human subjects research using specimens/ animals collected as part of the study, or those housed in archival collections. Examples include archaeology, paleontology, botany and zoology.**

Did the permission you obtained from a local authority to perform the study include an agreement on access to outputs and benefit sharing? This may include procedures to enable fair distribution of the benefits and resources arising from the research performed. Please include any details of Prior Informed Consent and Benefit Sharing Agreements obtained. These may be required by field-specific regulations, for example the Convention on Biological Diversity (CBD) and the associated Nagoya Protocol.

The permission we obtained from the local authority to perform the study did not include a formal agreement on access to outputs and benefit sharing. We regularly share our results with French Polynesian government authorities, French Polynesian scholars, and the local Nuku Hiva community through publications, talks, museum exhibit contributions, and other means.

If the material used in your study was imported, please A) provide the year it was imported and B) indicate whether permits were obtained to import/export the materials used, C) provide details of any permits obtained. If this information is not available, please indicate this.

The material used in this study was imported to New Zealand in 2011. Permits to export the material were provided by the Gouvernement de la Polynésie Française, Ministère de la Culture et de l'Artisanat, aided by the Service de la Culture et du Patrimoine (permit No. 1079). On importation to New Zealand, the materials were inspected, treated as needed, and released by the New Zealand Ministry of Agriculture and Forestry (now Ministry for Primary Industries).

If you used archival specimens, please state how the material used in your study was acquired by the institute it is held in and provide details of any permits obtained for the original excavations/ sample collection. If this information is not available, please indicate this.

The materials were originally excavated by Melinda Allen under permit No. 1079, Gouvernement de la Polynésie Française, Ministère de la Culture et de l'Artisanat, aided by the Service de la Culture et du Patrimoine. Plant and arthropod specimens recovered from the Ho‘oumi bulk samples are housed at the Deakin University, Earth Sciences and School of Life and Environmental Sciences Laboratories (Australia) respectively. Additional macroplant remains obtained in excavation are housed at the University of Auckland, School of Social Sciences, Roger C. Green Archaeological Laboratory (New Zealand). The bulk sediment samples were shipped to Australia for analysis following biosecurity procedures and permits of the two countries that were in place at the time.

How was the potential cultural significance of the materials collected in your study to local communities considered in your research design? Were Indigenous peoples and/or local researchers and institutions involved with archaeological excavations / collection of specimens? If so, please provide a description of their involvement.

The potential cultural significance of the materials collected from the larger Ho’oumi Valley study to local communities was considered in the research design. Marquesan community members were involved in obtaining permissions to carry out the research, both in advance of the study and on the ground. Local community members were also employed as field assistants. Individuals and their roles are identified in the Acknowledgements. The Covid-19 global pandemic has prohibited travel to the Marqquesas for the last two years. Although we have not yet had the opportunity to share the specific results reported in this article in person, we have a long-standing relationship with the local community and have in the past shared results through the local museum, with local school groups, through presentations, and through dissemination of published works.

If your manuscript includes photographs of human remains please indicate whether authors obtained permission from descendants or affiliated cultural communities to do so.

No human remains were recovered in this study.
